# Genomic catastrophes frequently arise in esophageal adenocarcinoma and drive tumorigenesis

**DOI:** 10.1038/ncomms6224

**Published:** 2014-10-29

**Authors:** Katia Nones, Nicola Waddell, Nicci Wayte, Ann-Marie Patch, Peter Bailey, Felicity Newell, Oliver Holmes, J. Lynn Fink, Michael C.J. Quinn, Yue Hang Tang, Guy Lampe, Kelly Quek, Kelly A. Loffler, Suzanne Manning, Senel Idrisoglu, David Miller, Qinying Xu, Nick Waddell, Peter J. Wilson, Timothy J.C. Bruxner, Angelika N. Christ, Ivon Harliwong, Craig Nourse, Ehsan Nourbakhsh, Matthew Anderson, Stephen Kazakoff, Conrad Leonard, Scott Wood, Peter T. Simpson, Lynne E. Reid, Lutz Krause, Damian J. Hussey, David I. Watson, Reginald V. Lord, Derek Nancarrow, Wayne A. Phillips, David Gotley, B. Mark Smithers, David C. Whiteman, Nicholas K. Hayward, Peter J. Campbell, John V. Pearson, Sean M. Grimmond, Andrew P. Barbour

**Affiliations:** 1 Queensland Centre for Medical Genomics, Institute for Molecular Bioscience, The University of Queensland, St Lucia, Brisbane, Queensland 4072, Australia.; 2 QIMR Berghofer Medical Research Institute, Herston, Brisbane, Queensland 4006, Australia.; 3 Surgical Oncology Group, School of Medicine, The University of Queensland, Translational Research Institute at the Princess Alexandra Hospital, Woolloongabba, Brisbane, Queensland 4102, Australia.; 4 Department of Anatomical Pathology, Princess Alexandra Hospital, Woolloongabba, Brisbane, Queensland 4102, Australia.; 5 The University of Queensland, UQ Centre for Clinical Research, Herston, Brisbane, Queensland 4029, Australia.; 6 The University of Queensland, School of Medicine, Herston, Queensland 4006, Australia.; 7 Flinders University Department of Surgery, Flinders Medical Centre, Bedford Park, South Australia 5042, Australia.; 8 St Vincent’s Centre for Applied Medical Research, University of Notre Dame and University of New South Wales, Sydney, New South Wales 2011, Australia.; 9 Cancer Research Division, Peter MacCallum Cancer Centre, East Melbourne, Victoria 3002, Australia.; 10 Department of Surgery, School of Medicine, The University of Queensland, Princess Alexandra Hospital, Woolloongabba, Brisbane, Queensland 4102, Australia.; 11 Cancer Genome Project, Wellcome Trust Sanger Institute, Hinxton, Cambridgeshire CB10 1SA, UK.; 12 Wolfson Wohl Cancer Research Centre, Institute for Cancer Sciences, University of Glasgow, Garscube Estate, Switchback Road, Bearsden, Glasgow G61 1BD, UK.

## Abstract

Oesophageal adenocarcinoma (EAC) incidence is rapidly increasing in Western countries. A better understanding of EAC underpins efforts to improve early detection and treatment outcomes. While large EAC exome sequencing efforts to date have found recurrent loss-of-function mutations, oncogenic driving events have been underrepresented. Here we use a combination of whole-genome sequencing (WGS) and single-nucleotide polymorphism-array profiling to show that genomic catastrophes are frequent in EAC, with almost a third (32%, *n* = 40/123) undergoing chromothriptic events. WGS of 22 EAC cases show that catastrophes may lead to oncogene amplification through chromothripsis-derived double-minute chromosome formation (*MYC* and *MDM2*) or breakage-fusion-bridge (*KRAS, MDM2* and *RFC3*). Telomere shortening is more prominent in EACs bearing localized complex rearrangements. Mutational signature analysis also confirms that extreme genomic instability in EAC can be driven by somatic *BRCA2* mutations. These findings suggest that genomic catastrophes have a significant role in the malignant transformation of EAC.

Oesophageal adenocarcinoma (EAC) has one of the poorest outcomes of all solid tumours, with only 14% of patients surviving 5 years^1^. Surgery remains the main curative treatment but it is only suitable for ~50% of patients due to the majority of EAC patients being diagnosed at an advanced stage of the disease^2^. Large-scale genomic studies, predominantly involving exome sequencing recently identified 26 genes significantly mutated in EAC^3,4^. Curiously, these genes were affected by widespread of loss-of-function mutations in tumour suppressors (*TP53, ARID1A, SMAD4*) whereas no obvious oncogenic mutations linked to EAC progression were identified.

The most significant risk factor for EAC is Barrett’s oesophagus (BE), a pre-malignant lesion that progresses into EAC in 5–10% of cases^2^. EACs can arise rapidly in patients diagnosed with BE, even in those under careful surveillance. It is currently believed that transformation of non-dysplastic BE to dysplastic BE and then on to cancer is driven by a stepwise accumulation of mutations but Weaver *et al*.^4^ showed that the majority of recurrently mutated genes in EAC can also be found in nondysplastic BEs that did not progress towards cancer. Only the tumour suppressor genes *TP53* and *SMAD4* have been shown to occur in a disease stage specific manner but these were unable to differentiate high-grade dysplastic BE from EAC. Taken together, these data suggest that oncogenic events underlying EAC progression are still at large.

While the mechanisms underlying EAC transformation are poorly understood, both telomere shortening and copy number alterations (CNAs) have been previously implicated^5–8^. Telomere shortening was observed in BE at all histologic grades^9^ and was associated with an increased risk of developing EAC^10^. Li *et al*.^2^ in a longitudinal study of BE-EAC progression showed that the genomes of BE that progress to EAC acquire significant increase of gain and losses with genomic diversity within 4 years of EAC diagnosis suggesting that a rapid genomic evolution may underlie the rapid EAC progression.

The present study addresses what role genomic rearrangements have in promoting EAC. A combination of WGS, single-nucleotide polymorphism (SNP) arrays and copy number analysis confirms that EACs have highly mutated and rearranged genomes^3,11^ and surprisingly that chromothripsis and breakage-fusion-bridge are frequent events in this cancer. Furthermore, these genomic catastrophes may underlie mechanisms of amplification of potent oncogenes through the formation of double-minute chromosomes and focal amplifications caused by breakage-fusion-bridge (BFB). Finally, this study suggests possible mechanisms for the rapid somatic genomic evolution that is a characteristic of this disease.

## Results

### Somatic mutations in EAC

In an effort to summarize the complete repertoire of somatic damage accumulated in EAC, a discovery cohort of 22 fresh-frozen EAC and matched normal tissues from EAC patients (all untreated before surgery) underwent WGS. The clinical features of the cohort disease were typical for EAC and are summarized in Supplementary Data 1. Analysis using qpure tool^12^ confirmed a median tumour cellularity of 73% and deep WGS was performed at an average base pair depth of 74-fold for tumour and 39-fold for normal samples (Supplementary Data 2). Somatic substitutions were identified using a dual-tool strategy using GATK^13^ and qSNP^14^, while indels were identified using Pindel^15^. Re-sequencing of one tumour/normal pair confirmed the accuracy of substitution calls to be 98.8%. Indels in coding regions were independently verified by amplicon deep sequencing, confirming 95% of the indels tested (Supplementary Data 3). CNA analysis was performed on high-density arrays using genome alteration print (GAP)^16^. Structural variants (SVs) were detected using the qSV tool. SV events were considered verified if presenting multiple lines of evidence in the WGS data and/or supported by boundaries of CNAs detected by SNP arrays, confirming 69% of the events (Supplementary Data 4).

Across the cohort, a total of 648,838 somatic SNVs and indels were identified. The median mutational burden genome-wide was 8.0 mutations/Mb (range of 1.53–34.56) (Supplementary Data 2 and 3). In coding regions, a total of 4,412 SNVs and indels were identified representing a median of seven mutations/Mb. These values are similar to previous reports^3,11^ placing EAC as one of the most mutated cancers along with bladder, colorectal, lung and melanoma^11^. In total, 434 genes harboured non-silent mutations in two or more patients (Supplementary Data 5). The overall mutation pattern closely resembled those previously reported by recent EAC exome sequencing efforts^3,4^, with 19 of the 26 significantly mutated EAC genes^3^ mutated in the present cohort (Supplementary Data 5). Copy number analysis identified 173 and 37 genes with recurrent gain or loss, respectively (Supplementary Data 6), many of which have been previously reported amplified (*CCNE1* (*n* = 7), *ERBB2* (*n* = 5), *FRS2* (*n* = 4), *GATA4* (*n* = 4), *KRAS* (*n* = 6), *MTMR9* (*n* = 6) and *MDM2* (*n* = 3)) or lost (*CDKN2A* (*n* = 17), *FHIT* (*n* = 8) and *RUNX1* (*n* = 13)) in EAC^5,6^.

### Mutational processes active in EAC

**Figure 1:**
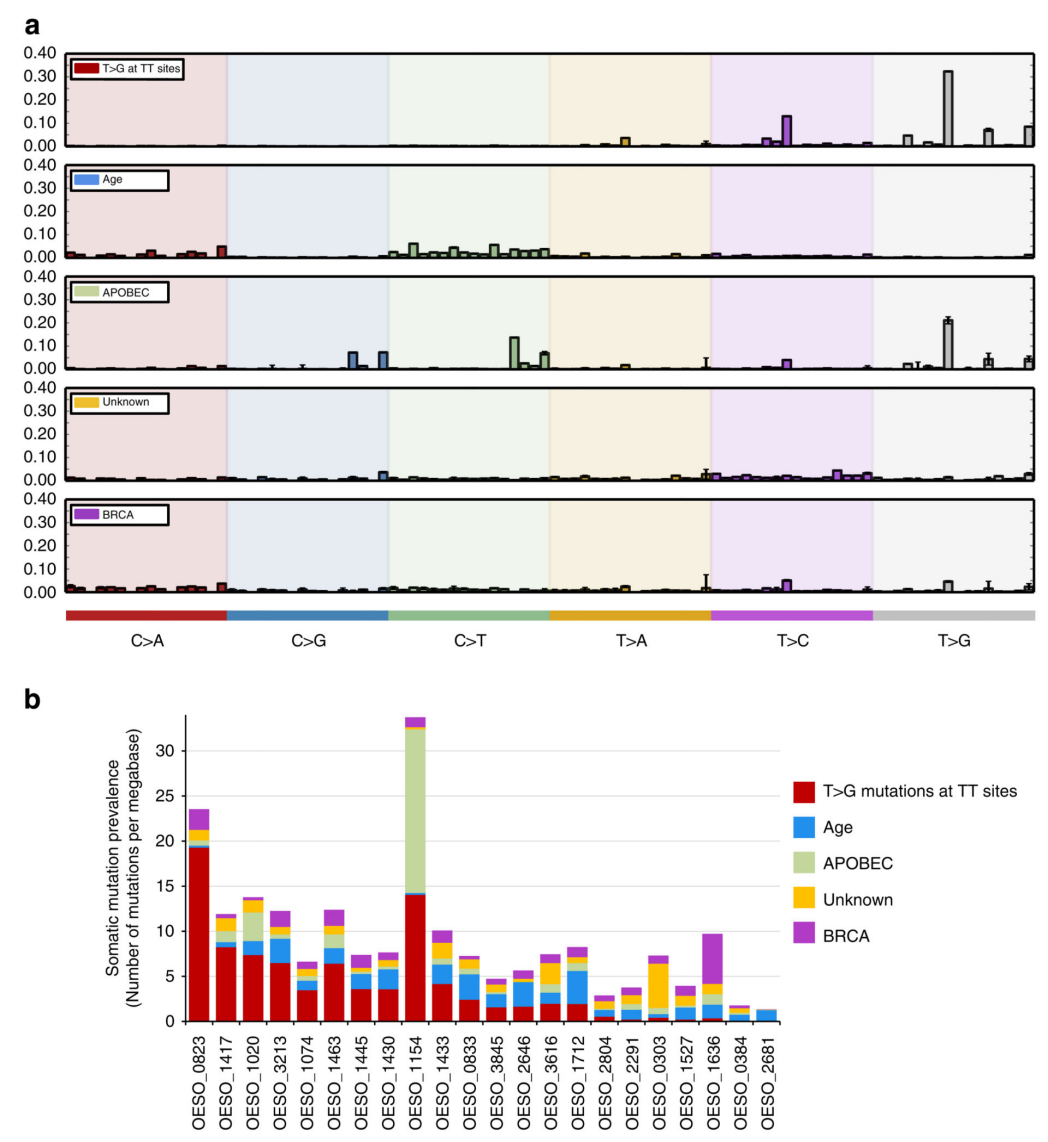
Mutational signatures found in EAC (**a**) Five mutational signatures were detected in EAC. Each signature is represented by the proportion of somatic substitutions (C>A, C>G, C>T, T>A, T>C and T>G). Substitutions are displayed in a trinucleotide context (including information about the bases immediately 3′ and 5′ to the mutated base) resulting in 96 potential contexts. (**b**) Contribution of mutational signatures operative in individual tumours. Each bar represents a tumour and the *y* axis represents the contribution of each signature within tumours, shown as number of mutations per Mb. The BRCA, the unknown and APOBEC signatures were most prevalent only in one tumour each (BRCA contributed ~57% of OESO_1636 mutations and unknown signature represents 67% of OESO_0303 mutations). The APOBEC signature, previously described in EAC and other tumour types^11^, contributes to more than 50% of the mutations in tumour OESO_1154, with small contributions in other samples. The age signature, previously described in EAC, is the second major operative mutational processes in this cohort. The signature characterized by T>G at TTsites is the most prominent within this cohort, representing ≥40% of mutations in 10 of 22 tumours (Supplementary Fig. 1).

Mutation spectral analysis confirmed that EACs display a preponderance of C>T transitions in coding sequences and T>G transversions across the genome^3,17^ (Supplementary Data 7). The sequence context of mutations can reveal mutational processes or signatures within tumours^11^. Three recently reported EAC mutational signatures^11^ were also detected here: Age signature (driven by general deamination), APOBEC signature and T>G mutations at TT sites. In addition, the BRCA-deficiency signature previously reported only in breast, ovarian and pancreatic tumours^11^ and an unknown signature were also observed (Fig. 1a). The signature characterized by T>G mutations at TT sites was the most prominent process within the cohort (Fig. 1b) and tumours with high contribution of this signature showed a trend towards poor survival (Supplementary Fig. 1). Strand bias was observed only for the signature characterized by T>G mutations at TT sites (Supplementary Fig. 2). T>G mutations at TT sites have been proposed to arise from oxidative DNA damage^3^. Reflux of gastric and bile acids which are risk factors for BE and EAC, have been associated with oxidative stress and oxidative DNA damage^18^. Furthermore incubation of BE tissues with a low pH bile acid cocktail leads to increased formation of 8-OH-dG^18^, which was suggested to be associated with T>G mutations at TT sites^19,20^. This might indicate a mechanism underlying this mutational signature that needs further investigation.

Localized hypermutation (kataegis) characterized by clusters of C>T and C>G mutations on the same strand^21^, was described here for the first time in EAC, with four tumours possessing numerous (≥10) kataegic foci (Supplementary Fig. 3) and 15 other EACs displaying occasional (<10 foci) kataegic regions (Supplementary Data 8). Consistent with previous studies implicating APOBEC proteins in kataegis^21,22^, the tumour with the highest number of kataegic regions (*n* = 17; OESO_1154) also had the highest contribution of the APOBEC signature (Fig. 1b).

### Subtyping of EAC using structural rearrangement patterns

SV analysis confirmed that EAC genomes are complex and highly-rearranged. A total of 7,343 SVs were identified (median of 263 SVs per tumour, range 126–776) (Supplementary Data 2 and 4). Many of these SV events were predicted to cause a loss-of-function by disrupting coding sequence of 2,984 genes; 642 of which were disrupted in two or more tumours (Supplementary Data 4 and 9). A screen for gene fusion events across the cohort identified 159 in-frame unique fusion genes (Supplementary Data 4). This rate of fusion gene formation was similar to what is expected by chance and is considered unlikely to drive cancer biology^23^.

The genomic distribution of SVs revealed considerable intertumour heterogeneity, enabling the categorization of the cohort into: unstable genomes (tumours with ≥450 SVs, *n* = 6), scattered (<450 SV events evenly distributed across the genome, *n* = 2) and complex localized (with a concentration of SVs in a single or few chromosomes, *n* = 14; Supplementary Fig. 4). Tumour OESO_1636 displayed an unstable genome (776 SVs) and harboured a somatic nonsense *BRCA2* mutation. The pathogenicity of this event was supported by a prominent BRCA mutational signature in this tumour (Fig. 1), suggesting an underlying mechanism previously implicated in genomic instability in other cancer types^24^.

### Genomic catastrophes in EAC

**Figure 2:**
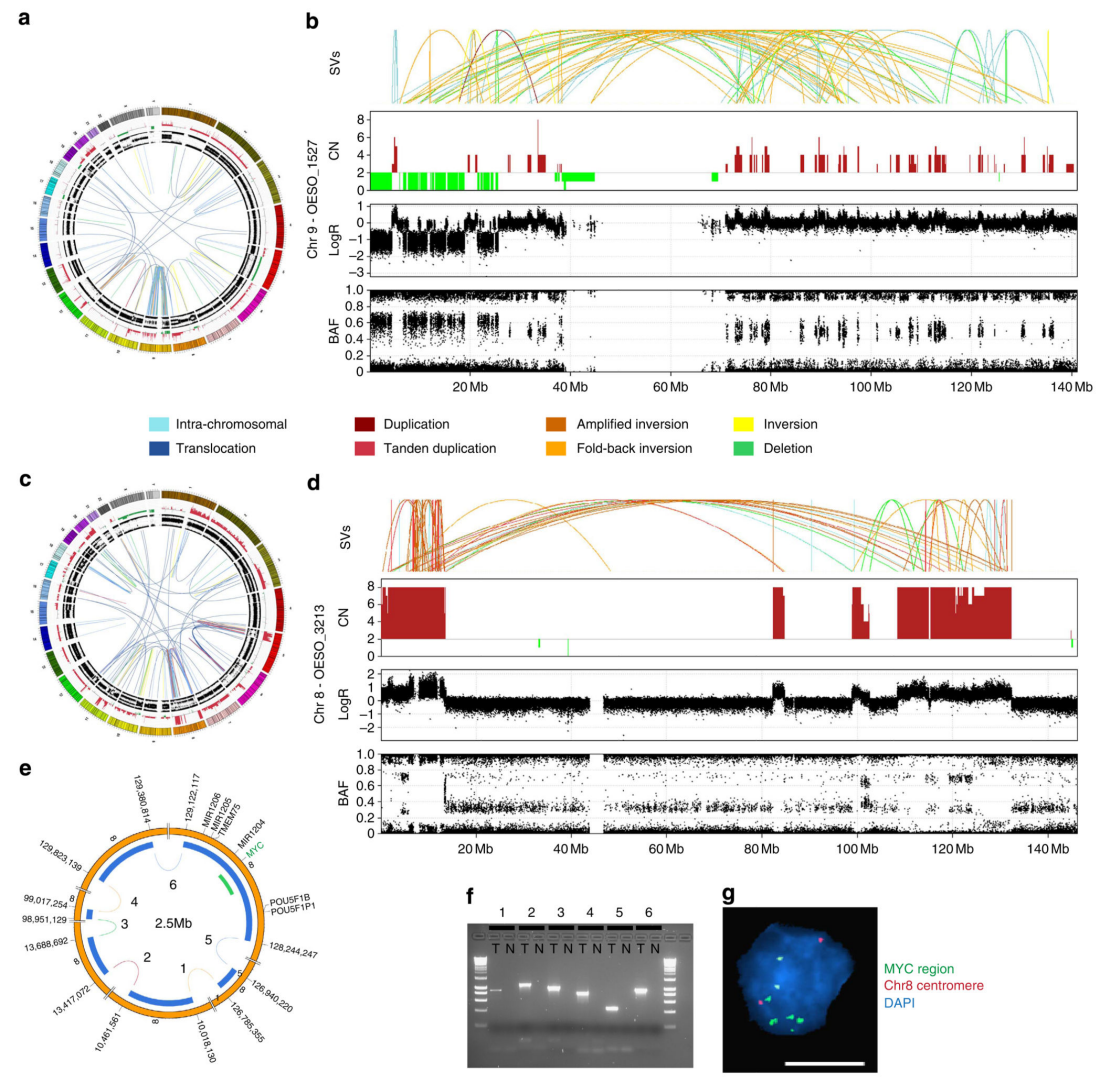
Evidence of chromothripsis in EAC tumours (**a**) Circos plot of tumour OESO_1527 containing copy number and BAF in the outer rings and somatic structural variants (SVs) are represented by lines in the inner ring. Colour of the lines represents SV type as indicated in the legend. Circos plot^37^ shows a high concentration of SVs on chromosome 9. (**b**) Zoomed-in view of events on chromosome 9 of tumour OESO_1527 showing evidence of chromosome shattering (chromothripsis). From top to bottom, graphs show SVs, copy number, logR ratio and BAF. There are changes in copy number state, concentration of a high number of SVs and retention of heterozygosity. (**c**) The genome distribution of somatic SVs for tumour OESO_3213. Circos plot containing copy number and SVs shows a concentration of events on chromosome 8. (**d**) Zoomed-in view of events on chromosome 8 in OESO_3213 showing SVs, copy number, logR ratio and B-allele frequency. (**e**) Inferred double-minute chromosome (DM) harbouring *MYC* oncogene. Blue blocks show fragments of chromosome 8 inferred to form DM and green block shows position of FISH probe. (**f**) Agarose gel showing PCR verification of SV events inferred to contribute to the DM. T, tumour, *N*, adjacent normal esophagus, numbers indicate SVs as shown in **e**. (**g**) FISH confirms copy number alterations in **d** by showing *MYC* amplification as multiple scattered signals and two copies of the chromosome 8 in a representative tumour cell nucleus. (Green—fluorescently labelled BAC RP11-367L7—*MYC* gene region; Red—centromeric region of chromosome 8—CHR8-10-RE). Scale bar, 10 μm. Supplementary Figures 11 and 12 are full images of gel and FISH presented in (**f**) and (**g**), respectively.

**Figure 3:**
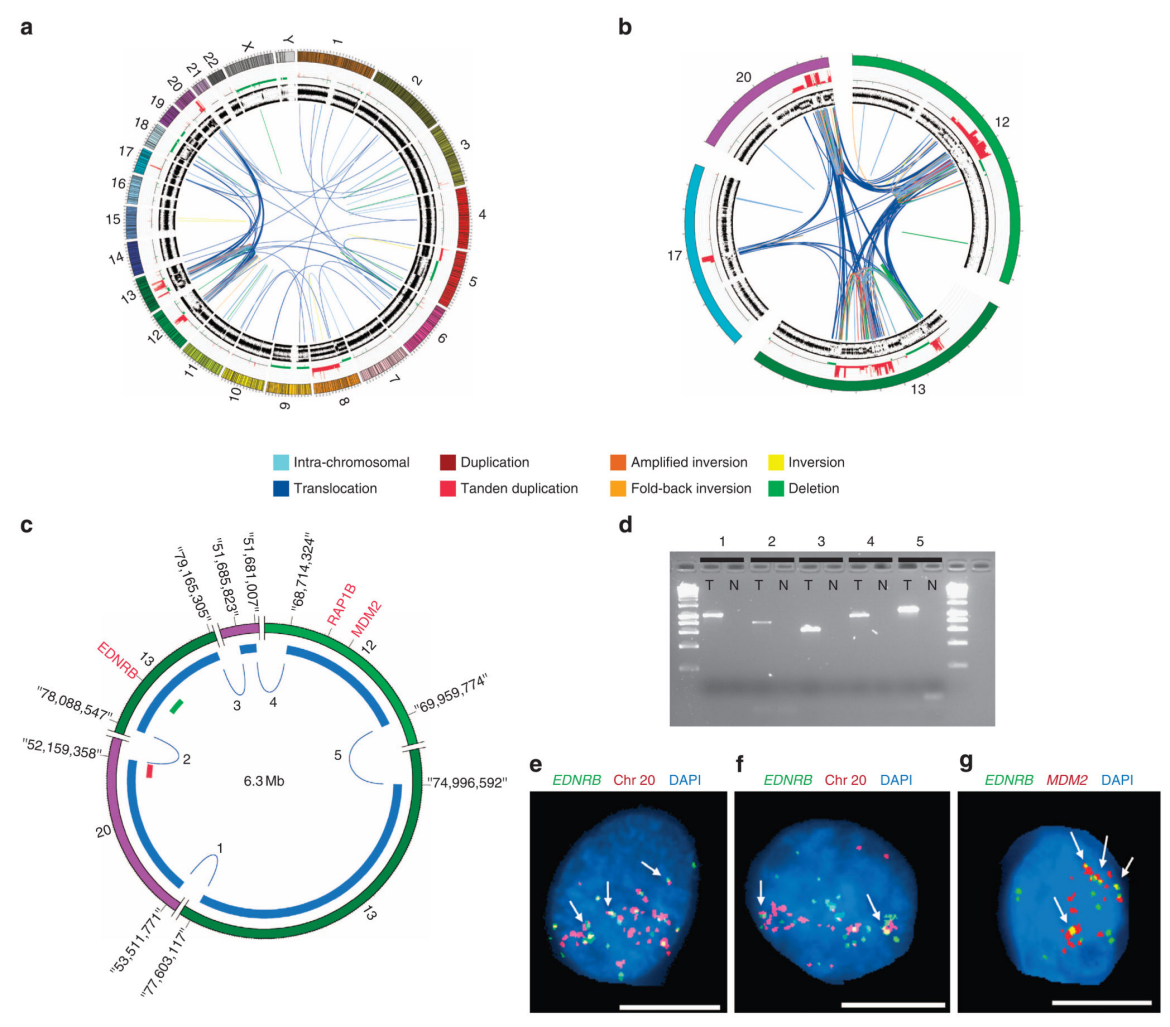
Evidence of a chromothriptic event involving four chromosomes (**a**) Overview of the distribution of events in the genome of tumour OESO_0384. Circos plot of tumour OESO_0384 containing copy number and B-allele frequency (outer rings) and somatic structural variants (SVs) are represented by lines in the inner ring. Colour of the lines represents SV type as indicated in the legend. (**b**) Detailed view of chromosomes 12, 13, 17 and 20 involved in the complex localized event. (**c**) Inferred DNA double-minute chromosome (DM) involving regions of chromosomes 12, 13 and 20. Red and green bars indicate location of FISH probes shown in **e**. (**d**) Agarose gel showing PCR verification of SV events inferred to be part of the DM in **c**. T, tumor, *N*, adjacent normal oesophagus, numbers indicate SVs as labelled in **c**. (**e,f**) FISH analysis demonstrated amplification of the two regions tested in representative tumour cell nuclei, plus the frequent colocalization of signals indicating fusion of regions of chromosomes 13 and 20 (see arrows for examples). Green—RP11-122N18 (chr13:78,416,550-78,591,831) to the *EDNRB* gene region, Red—RP11-192K14 (chr20:52,178,644-52,324,774). (**g**) FISH images show amplification and frequent colocalization of signals (see arrows for examples) indicating that chromosome regions of chr 13 and chr 12 are part of the inferred DM. FISH Green—RP11-122N18 (chr13:78,416,550-78,591,831) to *EDNRB* gene region and Red—RP11-77H17- (chr12:69,154,590-69,318,752) to *MDM2* gene region. Scale bar, 10 μm. Supplementary figures 13 to 16 are full images of Figure 3(**d**) to (**g**).

**Figure 4:**
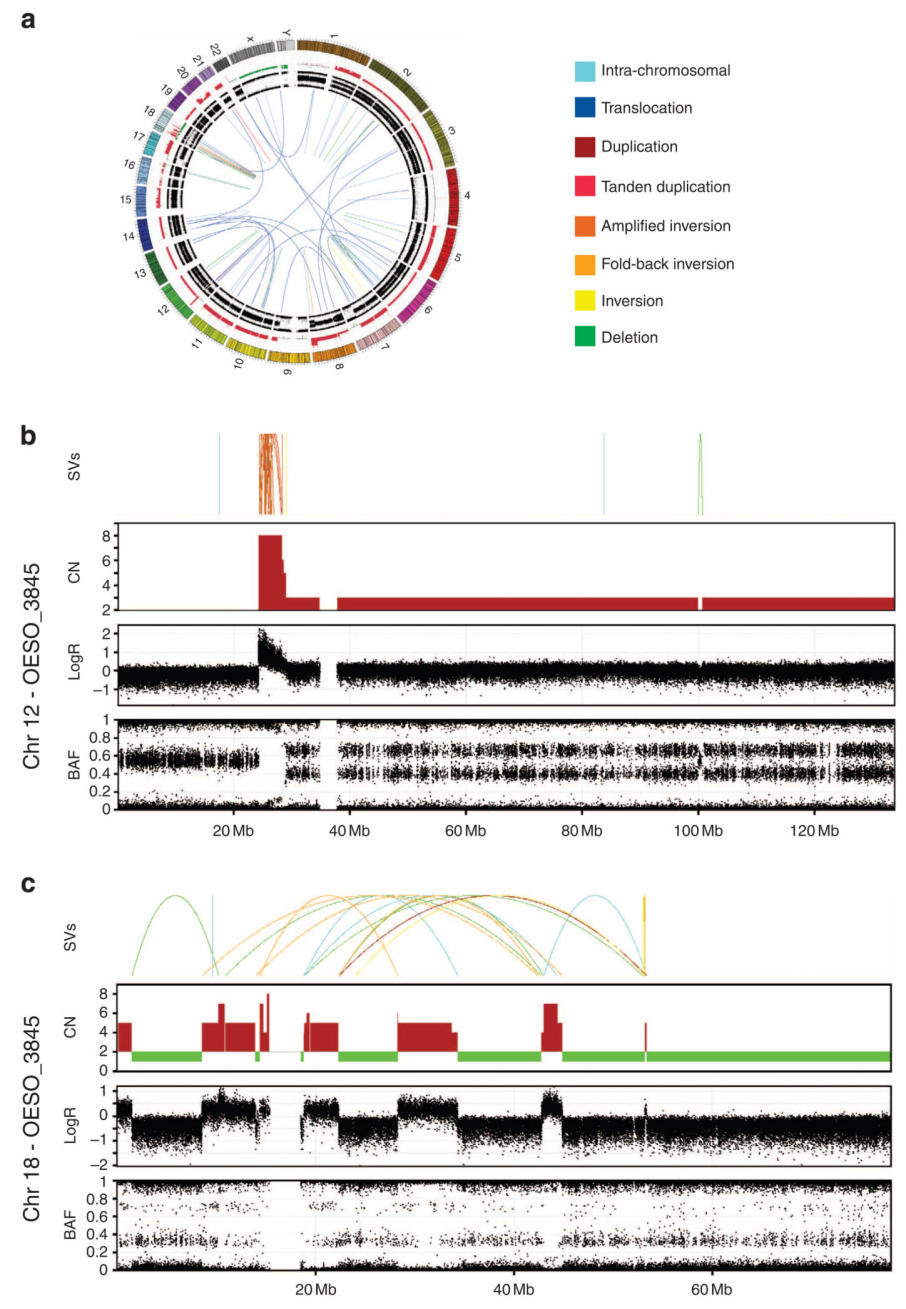
Chromothripsis and breakage-fusion-bridge (BFB) evidence in an EAC tumour OESO_3845 contained a high number of SVs concentrated in two chromosomes, 12 and 18. (**a**) Circos plot shows overall distribution of SVs in the genome. Circos plot containing copy number and B-allele frequency in the outer rings and somatic structural variants (SVs) are represented by lines in the inner ring. Colour of the lines represents SV type as indicated in the legend. (**b**) Zoomed-in view of events on chromosome 12. Graph shows from the top, SVs, copy number, logR ratio and B-allele frequency. Copy number profile suggests loss of telomeric p arm and SV events suggest several cycles of BFB on chromosome 12, with 20 inversions mapped to the amplified region (24,387,412 to 28,333,288 bp). (**c**) Zoomed-in view of events on chromosome 18. Graph shows SVs, copy number, logR ratio and B-allele frequency. SV events and copy number data suggests shattering of chromosome 18 with switches in copy number state, concentration of a high number of SVs and retention of heterozygosity characteristic of a chromothriptic event.

Chromothripsis has been reported in different cancer types^25–28^ and was initially thought to be present in 2 to 3% of tumours^25^. Zack *et al*.^29^ detected chromothripsis in 5% of tumours across 10 cancer types, ranging from 0 to 16%, however, higher frequency has been reported in bone cancer (25%)^25^ and medulloblastoma (36%)^27^. The prevalence of chromothripsis in cancer genomes has been recently reviewed by Kloosterman *et al*.^30^ This catastrophic event is a plausible explanation for the concentration of SVs in one or few chromosomes observed in EACs with complex localized genomes. Mitotic chromosome segregation errors that lead to chromosome shattering (chromothripsis)^25,31^ can affect several genes in only a few cell cycles and have the potential to drive cancer development^25^. Such events may represent an alternative mechanism to the stepwise accumulation of mutations that lead to malignant transformation. Surprisingly, eight of the 22 EACs (36%) in the discovery cohort contained rearrangements similar to chromothripsis (Figs 2–4, Supplementary Figs 5 and 6, Supplementary Data 10 and 11). This high frequency of chromothripsis was confirmed by screening a further 101 EACs using SNP arrays, with 32 out of 101 (32%) tumours showing ≥10 transitions between 2 or 3 copy number states, with loss and preservation of heterozygosity in one or few chromosomes (Supplementary Fig. 7). In total, 40 EACs out of 123 tumours (32.5%) showed evidence of chromothripsis.

It has been previously shown that a potential by-product of chromothripsis is the formation of double-minute chromosomes (DM)^25,27^. DMs may harbour oncogenes and if providing a competitive advantage to the cancer cells may be selected for further amplification during cell cycles resulting in a potential oncogenic event. Evidence of this driving mechanism was observed in half (*n* = 4) of the EACs with evidence of chromothripsis, these tumours showed high copy number of retained DNA fragments and SV events randomly connecting those regions of high copy number.

The tumour OESO_3213 has evidence of a 2.5 Mb DM derived from shattering of chromosome 8 and random fusion of six fragments; one of which harbours the *MYC* oncogene. The SV events inferred to be involved in the DM were validated by PCR. FISH analysis confirmed that gene amplification is not due to homogenously staining regions but extra chromosomal amplification of *MYC* gene (Fig. 2). In a second tumour (OESO_0384), evidence of DM arising from a chromothriptic event involving four chromosomes harboured *MDM2* gene was identified and similarly verified using PCR and FISH (Fig. 3). *MDM2* is an oncogene and known inhibitor of *TP53*.

In the discovery cohort of 22 EAC, six tumours displayed localized genomic damage consistent with BFB events (Fig. 4, Supplementary Fig. 8). BFB is known to begin with telomere loss, followed by fusion of unprotected chromosomal ends or sister chromatids fusion. These fused chromosomes are subsequently torn apart during anaphase. This process can be repeated for several cell cycles resulting in inverted duplications with dramatic copy number increases, and when such amplified regions harbour oncogenes, this may provide growth advantage to tumour cells. Regions amplified by BFB cycles in the discovery cohort harbour oncogenes or genes that promote tumour growth such as *RCF3, MDM2, VEGFA, BCAT1* and *KRAS*. Tumours with complex localized SVs in their genomes showed higher prevalence of inversions and deletions (*P*<0.05, *t*-test), with breakpoints presenting a high incidence of microhomology, suggesting microhomology-mediated break repair and/or non-homologous recombination as the major mechanism of DNA repair (Supplementary Fig. 9). WGS-based telomere integrity analysis found somatic telomere shortening in EAC, with tumours displaying complex localize rearrangements presenting a more prominent shortening (Supplementary Fig. 10).

Three EAC tumours showed evidence of both BFB and chromothripsis events (Supplementary Fig. 10). Dicentric chromosome formation by telomeric erosion has been suggested as a potential mechanism leading to chromothripsis as an extreme outcome of a mutagenesis mechanism^31^. Tumour OESO_3845 showed evidence of both BFB and chromothripsis (Fig. 4). Chromosome 12 showed a high concentration of SVs suggesting a series of BFB cycles between sister chromatids, with loss of the *p* arm telomeric region and 20 inversions mapped to a small amplified interval (Fig. 4). Approximately 30 genes are located in this amplified region, including the oncogene *KRAS* (wild type). In addition, the SV and copy number profile of chromosome 18 was indicative of chromothripsis, with lost regions harbouring several genes including tumour suppressor *SMAD4* (Fig. 4b). Amplification of the oncogene *KRAS* and loss of tumour suppressor *SMAD4* may fuel cancer progression in this tumour. Loss of *q* arm telomeric region of chromosome 18 with two inversions suggests evidence of BFB followed by chromothripsis.

Chromothripsis has recently been associated with *TP53* mutant medulloblastoma and AML^27^ and it has been suggested that chromothripsis occurs at higher frequencies in p53-deficient cells. In the present study 81% of the EACs harbour somatic *TP53* point mutations and an additional 9% of the tumours contained SV events that either inactivated *TP53* or led to *MDM2* gene amplification (Supplementary Data 4 and 12). The high frequency of *TP53* inactivation in EAC, together with widespread telomere shortening, may explain the high incidence of chromothripsis.

## Discussion

This is the first study to report evidence of chromothripsis in EAC and reveals that as many as a third of EAC genomes have undergone catastrophic chromosomal events. This is a much higher frequency than has been detected in other cancers (2–5%)^25,29^. Furthermore, our data suggest that large-scale chromosomal changes delivered by chromothripsis and BFB provide a mechanism for tumour suppressor gene loss or oncogene amplification, which may then be clonally expanded due to selective advantage, as reported in other cancer types^25,28^.

The observations reported here are important on two grounds. First, they provide an explanation for the paucity of oncogenic gain-of-function mutations identified in previous large-scale exome studies in EAC. Although cancers like melanoma or pancreatic adenocarcinoma are driven by point mutation of oncogenes *BRAF* and *KRAS*, respectively, EAC appears to obtain its oncogenic drive through amplification. Given the genome-wide distribution of catastrophic events seen in EAC, it is possible that this disease has the opportunity to use a broad range of oncogenes in transformation. Second, genomic catastrophes provide a plausible explanation for the ability of EAC to arise rapidly in patients, especially in those bearing BE. A catastrophe-driven transformation from BE-EAC hypothesis is further supported by previous studies showing that BE that progressed to EAC present increased somatic chromosomal alterations (gains and losses) with genomic diversity within 4 years before EAC diagnosis^2^, while BE that did not progress to EAC for a long time presented genomic stability. Telomere shortening and *TP53* mutation have been reported as early genomic events in BE^4,9^. These may represent a potential mechanism underlying genomic catastrophes in EAC, since cells lacking p53 enter mitosis prematurely with uncapped telomeres and those that persist in mitotic p53-deficient cells are shorter and prone to form end-to-end fusions^32^. Further studies using WGS of longitudinal cohorts of BE-EAC patients are required to strengthen the findings presented here.

Finally, this study highlights the value of interrogating tumours with a deep WGS approach when performing cancer-driver discovery studies. Genomic catastrophes, gene inactivation through chromosomal rearrangements and telomere integrity appear to be important in EAC, all of which would have been impossible to detect with exome-based cancer studies.

## Methods

### Samples

Samples used in this study are from patients that provided written informed consent, with approval from the Princess Alexandra Hospital research ethics committee (PAH-HREC-2007/068). Fresh-frozen tissue samples (snap frozen in liquid nitrogen) were obtained from the Princess Alexandra Hospital tumour bank. Clinical and pathological data were collected prospectively; tumours were assessed by an experienced gastrointestinal pathologist (G.L.) and classified on the basis of the current (7th) edition of the American Joint Committee on Cancer (AJCC) staging system. For each frozen sample, a 7-μm section was used to make a haematoxylin/eosin (H&E) slide, followed by two 30-μm sections for DNA extraction; this was repeated to obtain a minimum of six unstained sections with serial H&Es. The H&E slides were used to assess tumour percentage and guide macrodissection. Samples with a minimum of 50% tumour tissue as estimated by the pathologist were included in the study. DNA extraction was performed using the AllPrep DNA/RNA Mini Kit (Qiagen, Valencia, CA, USA). DNA was quantified using Qubit dsDNA BR Assay Kit (Invitrogen, Carlsbad, CA, USA). Cellularity of each tumour sample was estimated using SNP array data and qpure tool^12^ (Supplementary Data 2).

### SNP arrays and copy number analysis

Tumour and matched normal DNA was assayed with the Omini 2.5–8, V1.0—Illumina BeadChips as per manufacturer’s instructions (Illumina, San Diego CA, USA). SNP arrays were scanned on an iScan (Illumina) and data were processed using the genotyping module (v1.9.4) in GenomeStudio v2011.1 (Illumina) to calculate B-allele frequencies (BAF) and logR ratios. GAP^16^ was used to call somatic regions of copy number change—gain, loss or copy neutral LOH. SNP probes with low quality scores (GC score of <0.7) in the matched normal sample were removed thereby suppressing germline copy number events and probes that performed poorly. Segments were then classified as: amplified (copy number 6–8); gained (copy number 3–5); loss (copy number 0–1) or copy neutral LOH (copy number 2 with major allele contribution of 0 or 1). Commonly affected genes in regions with significant (q<0.05) gain and loss were determined using GISTIC v2.0 (ref. 33) and genes within regions of copy number change were annotated using ENSEMBL v70.

### Whole-genome sequencing

Sequence libraries are generated from 1 μg of genomic DNA using the standard library preparation technique using the Illumina TruSeq DNA Sample Preparation Kit (Catalogue #: FC-121-2001), on the basis of the protocol in the TruSeq DNA PCR-free sample preparation guide. This process comprises pre-fragmentation cleanup using paramagnetic sample purification beads (Agencourt AMPureXP reagents, Beckman Coulter), sample fragmentation and size selection targeting 300 bp inserts again by paramagnetic sample purification beads, then end-repair and final library size quality control and quantification. Paired-end reads of 101 bp were generated in a single lane of a HiSEQ v3 flowcell on the Illumina HiSEQ 2000 instrument (HiSEQ control software v1.5/Real Time Analysis 1.13) using TruSeq SBS Kit v3-HS (200 cycles, catalogue #: FC-401-3001). Sequencing was carried out by the Illumina Genome Network to a minimum average of 30-fold base coverage for normal samples and 60-fold coverage for tumour samples.

### Mutation calling

Paired-end sequencing reads were aligned to the human genome (NCBI build37) using multi-threaded BWA^34^ 0.6.2-mt with sorted lane level BAMs (compressed binary version of sequence alignment/map format) created by Samtools^35^ 0.1.19. Whole sample level merged BAMs for tumour and matched normal samples of each patient were produced by in-house tools and optical duplicate reads marked using Picard MarkDuplicates 1.97 (http://picard.sourceforge.net). These contained all mapped and unmapped data and included pair orientation. SNVs were identified using dual calling strategy: qSNP^14^ which uses heuristic approach and GATK^13^ which is a Bayesian caller. To maximize accuracy, SNPs identified by both callers were used as the ‘High Confidence’ calls, subsequently used in further analysis. The Pindel tool^15^ was used to identify small insertions and deletions. Output filtering was developed in house to identify somatic short insertions and deletions. Examination of the context sequence surrounding each variant was carried out, excluding indels adjacent to homopolymer regions or near simple repeats. The remaining indels were compared with our locally developed database of germline indels identified across diverse cancer samples. They are also required to have supporting reads on both strands and a minimum of 5% supporting reads from the total potential informative reads spanning the region. BAM files for normal and tumour samples have been submitted to European Genome-phenome Archive (EGAS00001000750).

### Mutational signature

Mutational signatures were deciphered using the framework developed by Alexandrov *et al*.^11^ (http://www.mathworks.com/matlabcentral/fileexchange/38724). Mutational signatures are displayed using a 96-context classification defined by the substitution class and the sequence context immediately 3′ and 5′ to the mutated base.

### Kataegis

To identify loci of localized hypermutation, termed kataegis, only high confidence somatic call (called by both GATK and qSNP) were used. For each sample, all mutations were ordered by chromosomal position and the intermutation distances were calculated as the number of base pairs from one mutation to the next. Intermutation distances were then segmented using piecewise constant fitting to find regions of constant intermutation distance^21^. Parameters used for piecewise constant fitting were c525 and kmin52 (ref. 21). Putative regions of kataegis were identified as those segments containing six or more consecutive mutations with an average intermutation distance of ≤1,000 bp.

### Structural variant analysis

Structural rearrangements were identified using the qSV tool (http://sourceforge.net/p/adamajava/wiki/qSV/)), which uses multiple lines of evidence to detect somatic events including discordant pairs, soft clipping and regional *de novo* contig assembly of unmapped reads across potential breakpoints. Calls were considered high confidence when events had at least two lines of evidence (discordant read pairs, split reads and/or soft clipping) or a single evidence source with at least 10 supporting reads and no evidence observed in the matched normal sample. Reads mapped to repeat region with high coverage (>1000) were removed from the high confidence events, which were used for further analysis. A further line of evidence was supplied by overlapping copy number segment boundaries (SNP arrays) with breakpoint positions. Breakpoints and copy number segments (GAP) that overlap in a range of ± 30,000 bp based were classified as: deletions, duplications, tandem duplications, foldback inversions, amplified inversions, inversions, intrachromosomal or translocations according to the read pair types supporting the event together with copy number segments (arrays). Centromeric and telomeric events (within 100 kb) and genes affected by breakpoints were annotated using ENSEMBL v70. The likely consequence of the rearrangement was determined from the nature of the rearrangement and the transcription direction of affected genes.

### Localized complex events

The number of SV events was estimated by qSV tool as described above (Supplementary Data 4). The number of double strand breaks per Mb in the genome and each chromosome were estimated for each tumour. If one or more chromosomes presented three times more breaks per Mb than expected and if breaks were equally distributed in the genome, they were classified as localized complex. Evidence of clustering of breakpoints was estimated as proposed by Korbel and Campbell^36^. Chromosomes with evidence of clustering of breakpoints (*P*<0.001, Kolmogorov–Smirnov test—goodness of fit test) were reviewed for: (1) evidence of chromothripsis which included oscillation of copy number, random joins and retention of heterozygosity and (2) evidence of BFB which included loss of telomeric region with neighbouring highly amplified region harbouring inversions. A larger cohort of EACs (*n* = 101) was screened for evidence of chromothripsis using SNP arrays (Illumina), chromothripsis was inferred in cases where one or few chromosomes showed at least 10 switches in copy number states, with retention of heterozygosity.

### Verification of somatic mutations

Primers for somatic indels (*n* = 188) were designed using qAmplicon (http://sourceforge.net/p/adamajava/wiki/qAmplicon/). We were able to design primer for 179 indels. PCRs were performed for tumour and matched normal pairs. PCR products were pooled by patient and tissue type. Each pool was then diluted in DNase/RNase-free water to a final concentration of 0.2 ng μl, then barcoded libraries were prepared for each pool using the Illumina Nextera XT DNA Sample Preparation Kit. Libraries were quantified on the Perkin Elmer LabChip GX, normalized and pooled together in equimolar ratio. The library pool was diluted and denatured according to the standard MiSeq protocol, and 11 pM was loaded for sequencing on a 2 × 301 bp run on the Illumina MiSeq using the 600 cycle MiSeq Reagent Kit v3. MiSeq data were mapped using BWA-MEM, and tumour and matched normal BAMs were manually reviewed in IGV. A total of 166 indels had sequence coverage >200 × for tumour and normal sample (those were considered testable) with a verification rate of 95%.

HiSeq X Ten data of a re-sequenced tumour matched normal pair (OESO_0384) was used for SNP verification. Sequencing was performed by the Garvan Center for Clinical Genomics using 100 ng of DNA and eight cycles of PCR. Fastq files were mapped with BWA-MEM. Substitutions were called verified if evidence of the somatic event was observed in the XTen data. A total of 5,480 SNPs were tested with a verification rate of 99.56%.

Structural variants suspected to be involved in DM formation were verified by PCR across the rearrangement’s breakpoint and confirmed by gel electrophoresis. A total of six events were validated for inferred DM on patient OESO_3212 and five events for OESO_0384 DM. PCR primers were designed using qAmplicon to span the predicted breakpoint. PCR reactions were carried out in the tumour and matched normal genomic DNA using a 25 μl reaction composed of 22 μl of Platinum Taq DNA polymerase (Invitrogen), 2 μl of 10 μM primer (Integrated DNA Technology) and 1 μl of genomic DNA as template (1 ng μl). The following parameters were used for PCR; initial denaturation at 94 °C for 2 min; followed by 35 cycles: denaturation at 94 °C 30 s; annealing at 60 °C 30 s; extension at 68 °C 1 min; followed by final extension at 68 °C for 15 min. PCR products were visualized by gel electrophoresis and if a PCR band of the expected size was observed only in the tumour and not in the normal, the somatic event was considered verified.

### Assessment of telomeric DNA content

Reads containing the telomeric repeat (5′-TTAGGG-3′) × 3 or (5′-CCCTAA-3′) × 3 were counted and normalized to the average genomic coverage (the average base coverage of each genome). The normalized telomere count was obtained separately for each tumour and its matching normal. Ratio was calculated by tumour normalized counts/normal normalized counts.

### Fluorescent *in situ* hybridization

Frozen sections of tumour OESO_0384 and OESO_3213 were cut in a cryostat at 5 μm thickness. Sections were fixed in ice cold 100% methanol for 2-1/2 min and air dried. Probes were applied to sections (0.5 μl of each probe in 5 μl hybridisation buffer, Empire Genomics) under coverslips. Probes were denatured at 83 °C for 3 min and incubated in a humid slide chamber overnight (~16 h) at 37 °C. Sections were washed as follows: 2 min at 73 °C in 0.4 × SSC/0.3% IGEPAL followed by 1 min at room temp in 2 × SSC/0.1% IGEPAL followed by three rinses in water. Slides were mounted in 0.2 mg ml 4′,6-diamidino-2-phenylindole in Prolong Gold Antifade reagent (Life Technologies). Slides were visualized using a Zeiss Axio Fluorescence microscope and images were captured using Applied Imaging Cytovision software (Applied Imaging International) on an Olympus BX51 Fluorescence microscope.

## Supplementary Material

Supplementary Figures 1-16

Supplementary data 1

Supplementary data 2

Supplementary data 3

Supplementary data 4

Supplementary data 5

Supplementary data 6

Supplementary data 7

Supplementary data 8

Supplementary data 9

Supplementary data 10

Supplementary data 11

Supplementary data 12

## References

[1] Eloubeidi MA, Mason AC, Desmond RA, El-Serag HB (2003). Temporal trends (1973-1997) in survival of patients with esophageal adenocarcinoma in the United States: a glimmer of hope?. Am. J. Gastroenterol.

[2] Li X (2014). Temporal and Spatial evolution of somatic chromosomal alterations: a case-cohort study of Barrett’s esophagus. Cancer Prev. Res. (Phila).

[3] Dulak AM (2013). Exome and whole-genome sequencing of esophageal adenocarcinoma identifies recurrent driver events and mutational complexity. Nat. Genet.

[4] Weaver JM (2014). Ordering of mutations in preinvasive disease stages of esophageal carcinogenesis. Nat. Genet.

[5] Goh XY (2011). Integrative analysis of array-comparative genomic hybridisation and matched gene expression profiling data reveals novel genes with prognostic significance in oesophageal adenocarcinoma. Gut.

[6] Frankel A (2014). Genome-wide analysis of esophageal adenocarcinoma yields specific copy number aberrations that correlate with prognosis. Genes Chromosomes Cancer.

[7] Shiraishi H (2009). Telomere shortening in Barrett’s mucosa and esophageal adenocarcinoma and its association with loss of heterozygosity. Scand. J. Gastroenterol.

[8] Shammas MA (2008). Telomere maintenance in laser capture microdissection-purified Barrett’s adenocarcinoma cells and effect of telomerase inhibition in vivo. Clin. Cancer Res.

[9] Finley JC (2006). Chromosomal instability in Barrett’s esophagus is related to telomere shortening. Cancer Epidemiol. Biomarkers Prev.

[10] Xing J (2009). Constitutive short telomere length of chromosome 17p and 12q but not 11q and 2p is associated with an increased risk for esophageal cancer. Cancer Prev. Res. (Phila).

[11] Alexandrov LB (2013). Signatures of mutational processes in human cancer. Nature.

[12] Song S (2012). qpure: a tool to estimate tumor cellularity from genome-wide single-nucleotide polymorphism profiles. PLoS ONE.

[13] McKenna A (2010). The genome analysis toolkit: a mapreduce framework for analyzing next-generation DNA sequencing data. Genome Res.

[14] Kassahn KS (2013). Somatic point mutation calling in low cellularity tumors. PLoS ONE.

[15] Ye K, Schulz MH, Long Q, Apweiler R, Ning Z (2009). Pindel: a pattern growth approach to detect break points of large deletions and medium sized insertions from paired-end short reads. Bioinformatics.

[16] Popova T (2009). Genome Alteration Print (GAP): a tool to visualize and mine complex cancer genomic profiles obtained by SNP arrays. Genome Biol.

[17] Agrawal N (2012). Comparative genomic analysis of esophageal adenocarcinoma and squamous cell carcinoma. Cancer Discov.

[18] Dvorak K (2007). Bile acids in combination with low pH induce oxidative stress and oxidative DNA damage: relevance to the pathogenesis of Barrett’s oesophagus. Gut.

[19] Inoue M (1998). Induction of chromosomal gene mutations in Escherichia coli by direct incorporation of oxidatively damaged nucleotides. New evaluation method for mutagenesis by damaged DNA precursors *in vivo*. J. Biol. Chem.

[20] Satou K (2009). Involvement of specialized DNA polymerases in mutagenesis by 8-hydroxy-dGTP in human cells. DNA Repair (Amst).

[21] Nik-Zainal S (2012). Mutational processes molding the genomes of 21 breast cancers. Cell.

[22] Taylor BJ (2013). DNA deaminases induce break-associated mutation showers with implication of APOBEC3B and 3A in breast cancer kataegis. Elife.

[23] Campbell PJ (2010). The patterns and dynamics of genomic instability in metastatic pancreatic cancer. Nature.

[24] Popova T (2012). Ploidy and large-scale genomic instability consistently identify basal-like breast carcinomas with BRCA1/2 inactivation. Cancer Res.

[25] Stephens PJ (2011). Massive genomic rearrangement acquired in a single catastrophic event during cancer development. Cell.

[26] Molenaar JJ (2012). Sequencing of neuroblastoma identifies chromothripsis and defects in neuritogenesis genes. Nature.

[27] Rausch T (2012). Genome sequencing of pediatric medulloblastoma links catastrophic DNA rearrangements with TP53 mutations. Cell.

[28] Li Y (2014). Constitutional and somatic rearrangement of chromosome 21 in acute lymphoblastic leukaemia. Nature.

[29] Zack TI (2013). Pan-cancer patterns of somatic copy number alteration. Nat. Genet.

[30] Kloosterman WP, Koster J, Molenaar JJ (2014). Prevalence and clinical implications of chromothripsis in cancer genomes. Curr. Opin. Oncol.

[31] Crasta K (2012). DNA breaks and chromosome pulverization from errors in mitosis. Nature.

[32] Thanasoula M (2010). p53 prevents entry into mitosis with uncapped telomeres. Curr. Biol.

[33] Mermel CH (2011). GISTIC2.0 facilitates sensitive and confident localization of the targets of focal somatic copy-number alteration in human cancers. Genome Biol.

[34] Li H, Durbin R (2009). Fast and accurate short read alignment with Burrows-Wheeler transform. Bioinformatics.

[35] Li H (2009). The Sequence Alignment/Map format and SAMtools. Bioinformatics.

[36] Korbel JO, Campbell PJ (2013). Criteria for inference of chromothripsis in cancer genomes. Cell.

[37] Krzywinski M (2009). Circos: an information aesthetic for comparative genomics. Genome Res.

