# Supplementary material for: Genomic catastrophes frequently arise in esophageal adenocarcinoma and drive tumorigenesis
Source: Nat Commun. Author manuscript; Available in PMC 2015 Oct 7. (PMC4596003; doi:10.1038/ncomms6224)
Supplement: Supplementary Figures 1-16 [file NIHMS64791-supplement-Supplementary_Figures_1-16.pdf]

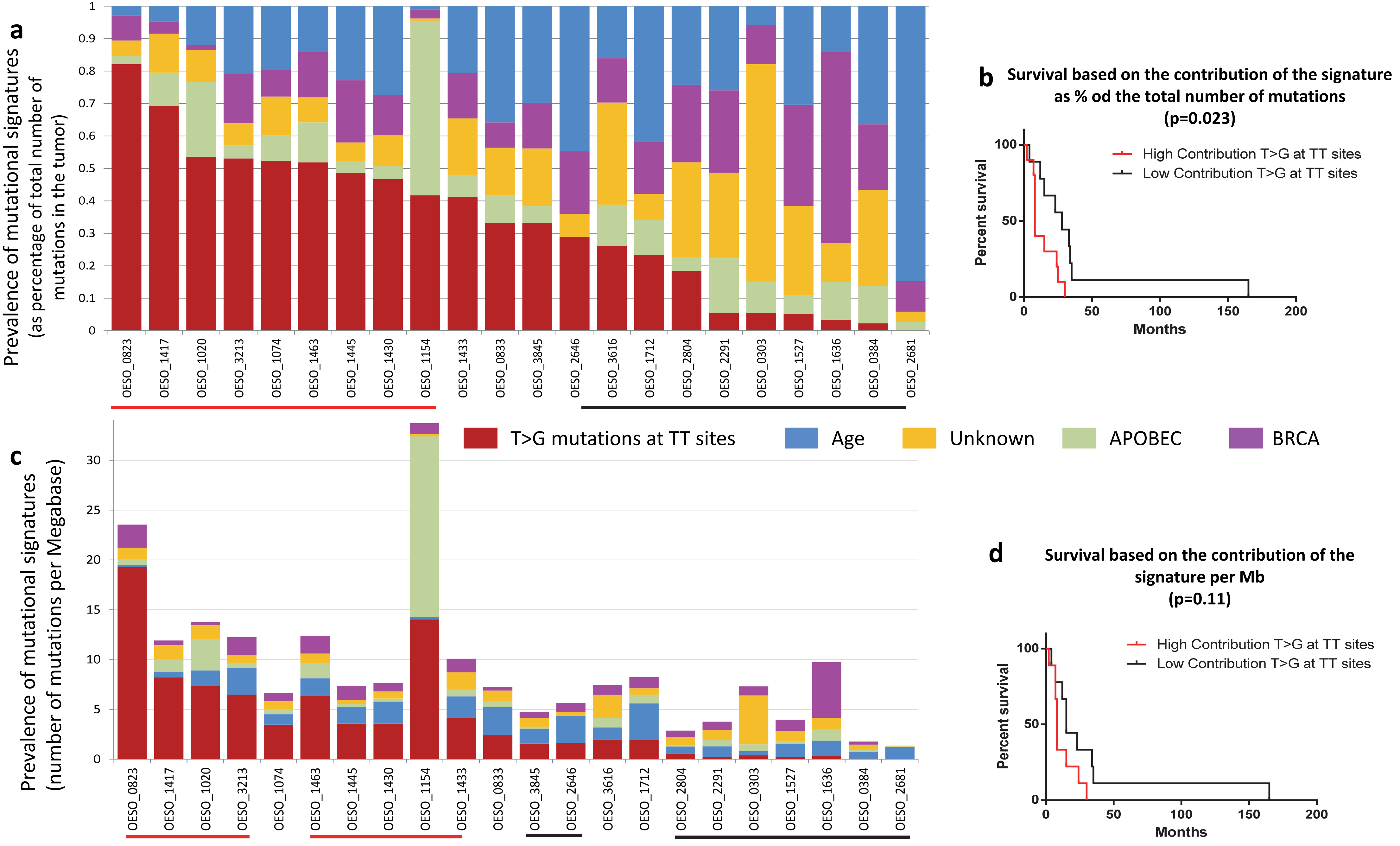

Supplementary Figure 1. Survival based on contribution of the signature characterized by T>G at TT sites. A) Prevalence of each signature per tumor (represented as a percentage of the total number of mutations in a particular tumor). Red line under the graph represent the top 40% tumors with higher contribution of T>G mutation at TT sites as percentage of the total number of mutations. Black line represents the 40% with lower contribution of this signature. B) Kaplan-Meier survival curves of patients whose contribution of the signature characterized by T>G at TT sites was classified as 40% highest and lowest contribution (Log-rank Martel-Cox test). C) Prevalence of the mutational signatures per Mb, tumors are presented in the same order of graph in section A. Red line under the graph represent the top 40% with higher contribution of T>G mutation at TT sites per Mb . Black line the 40% with lower contribution of this signature. D) Kaplan-Meier survival curves of patients whose contribution of the signature characterized by T>G at TT sites per Mb was classified as 40% highest and lowest contribution (Log-rank Martel-Cox test).

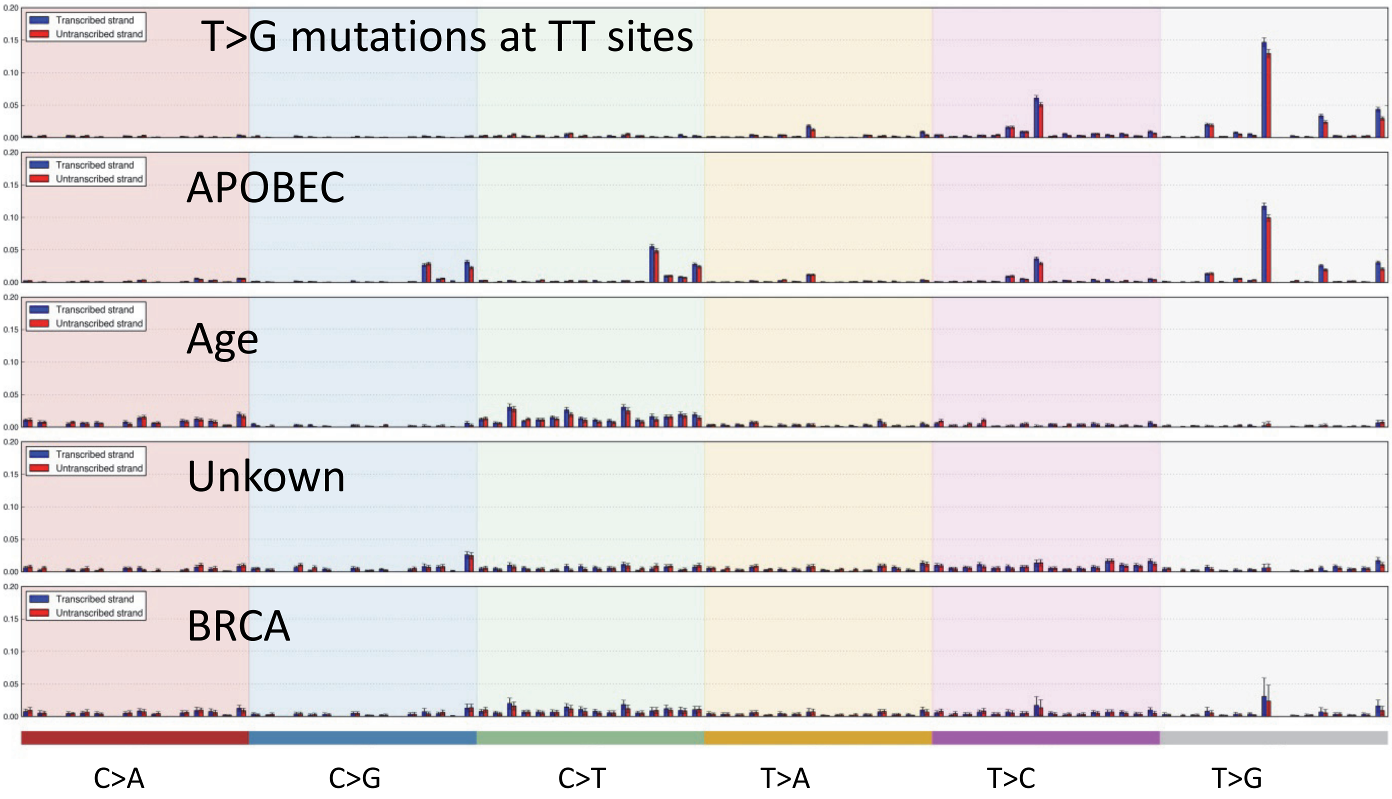

Supplementary Figure 2. Transcriptional strand of mutational signatures. Mutations are shown according to the substitution type and the sequence context immediately 5' and 3' to the mutated base. Only T>G at TT sites signature showed transcriptional strand bias. Mutation types in the x axis and percentage of mutations attributed to a specify mutation type in Y axis.

Four tumors with numerous (≥10) foci of kataegis

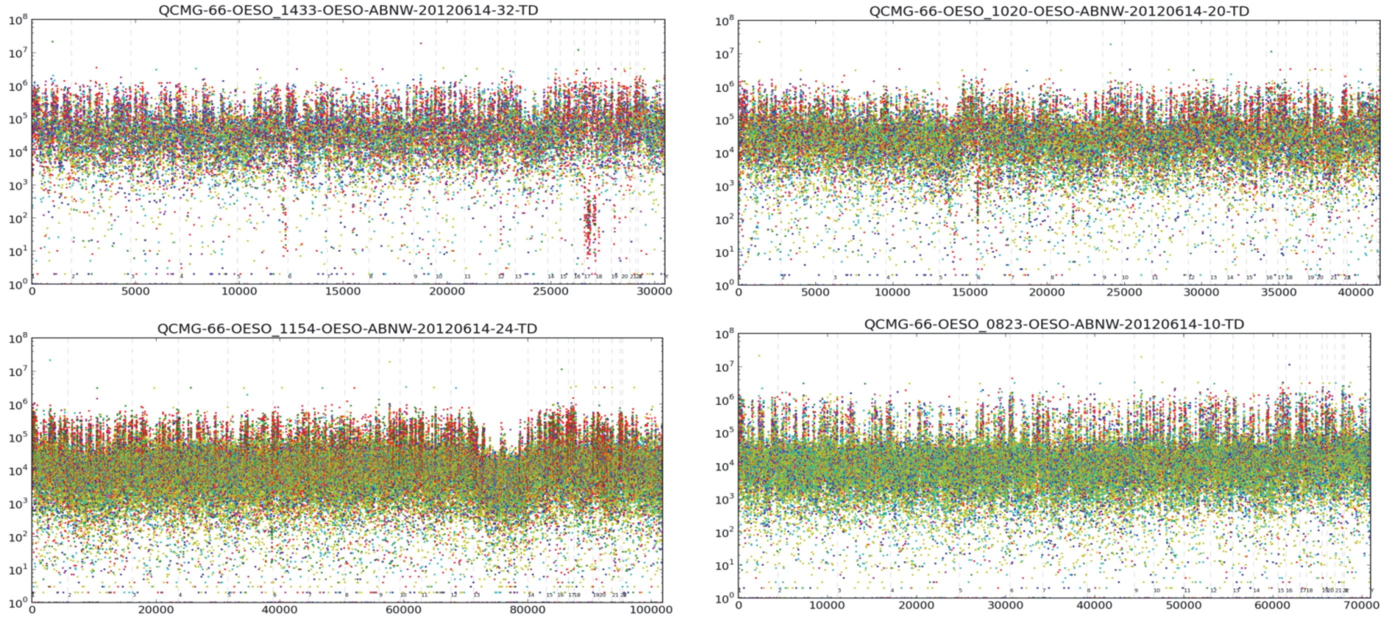

Three tumors did not show evidence of kataegis

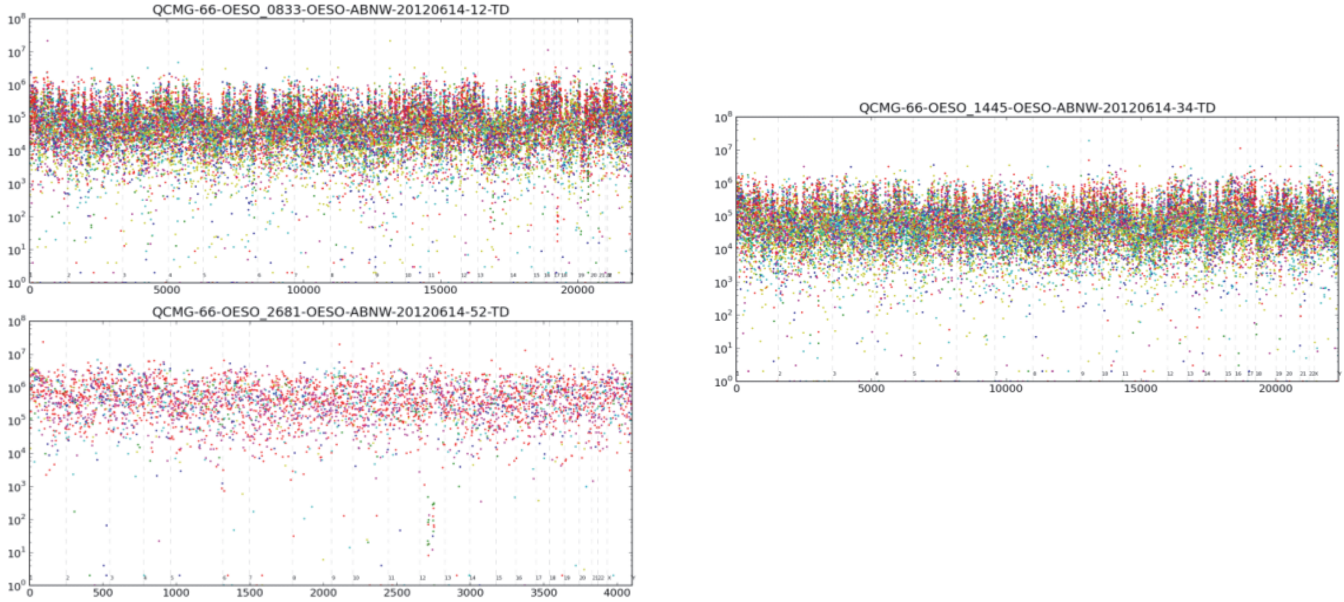

3 examples of the 15 OAC tumors that showed occasional (<10) kataegic foci

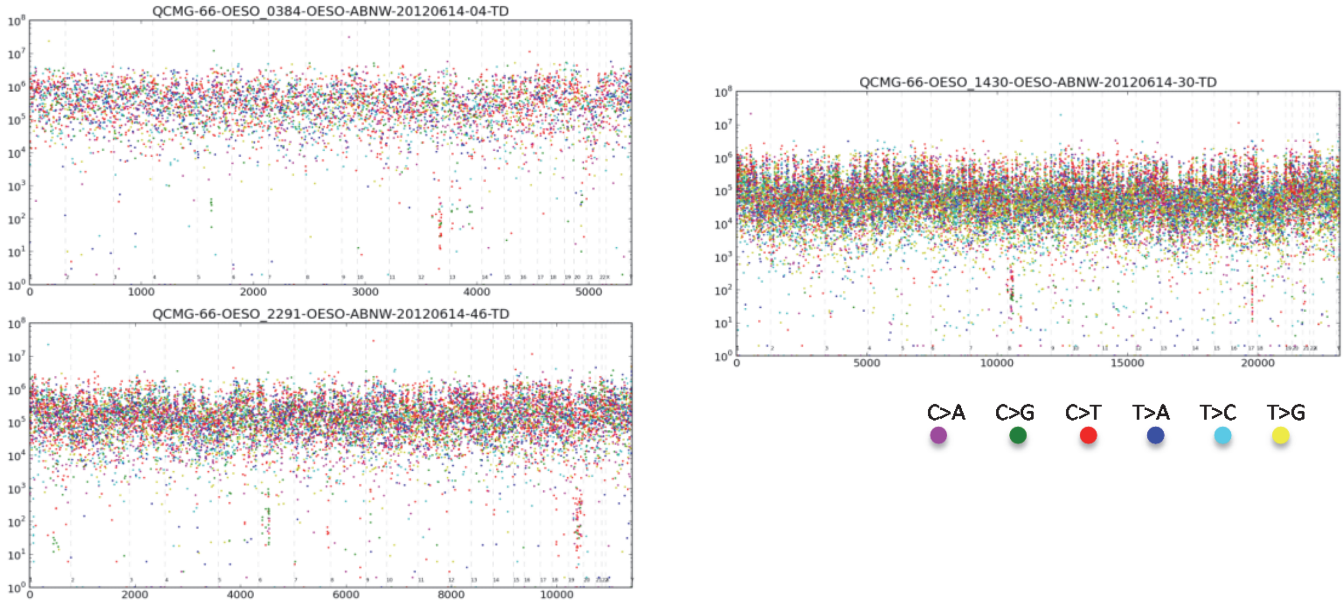

Supplementary Figure 3. Kataegis, regional hypermutation of base substitution**.** Rainfall plots . Intermutation distance (bp) is presented in the x axis and mutation number in the y axis.

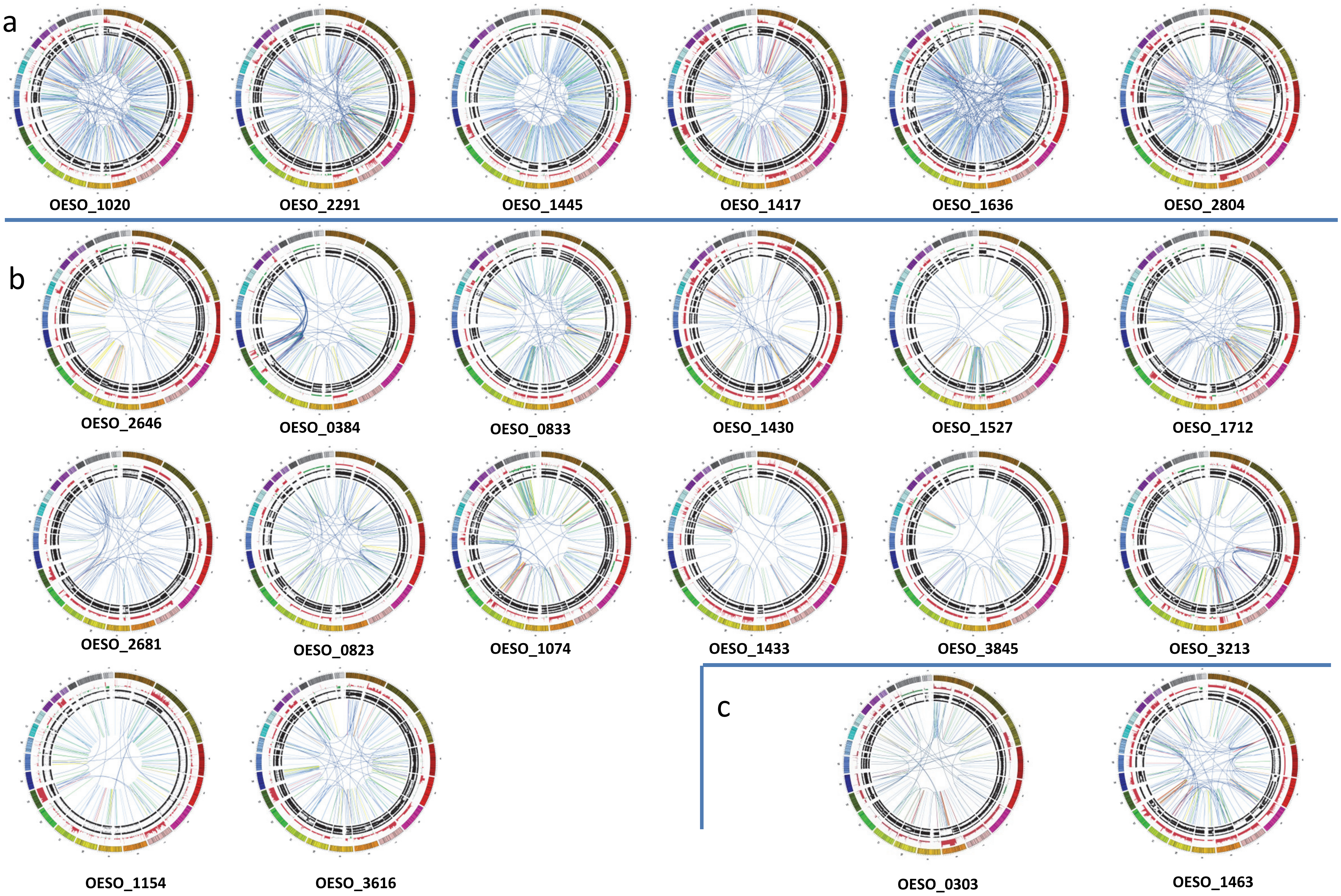

Supplementary Figure 4. Overview of structural variants distribution in the genome of each EAC tumor. Circos plot containing copy number and B allele frequency in the outer rings and somatic structural variants in the centre. Tumors were stratified into 3groups: A) *Unstable* (≥450 SV); B) *Complex Localized* (concentration of SVs in a single or few chromosomes) and C) *Scattered* (<450 SV events evenly distributed across the genome).

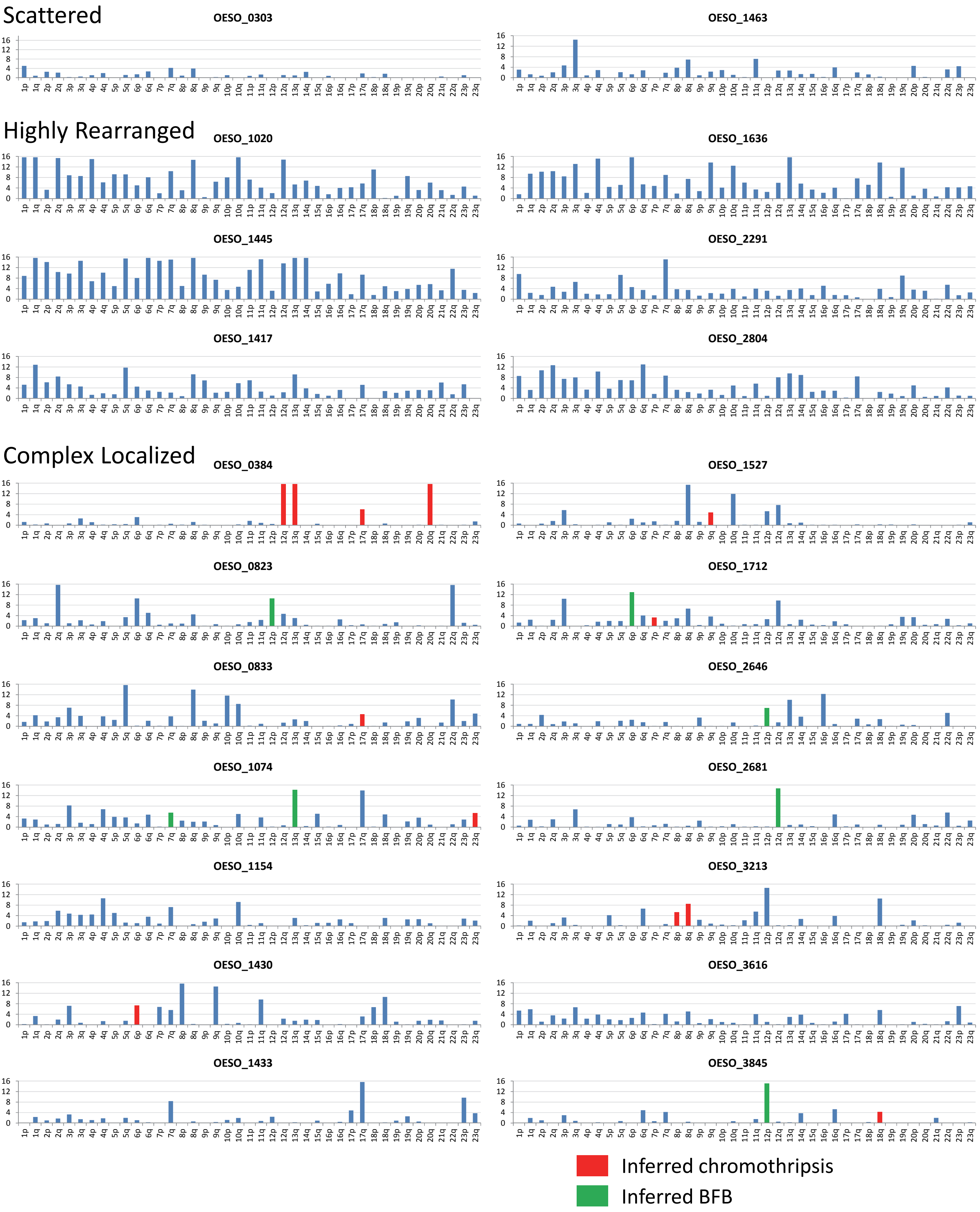

Supplementary Figure 5. Evidence of breakpoint clustering. Statistical significance for deviation from the null hypothesis (random breakpoint distribution) as proposed by Korbel and Campbell (2013). Chromosome arms are showed in the x-axis and –log10(p-values) in the y axis. Evidence of non-random breakpoint distribution was considered when p-value<0.001, Kolmogorov-Smirnov test – goodness of fit test. Chromosomes that pass this threshold were inspected for other criteria of chromothripsis (oscillation of CNA and retention of heterozygosity) and BFB (loss of telomeric region, amplification with concentration of inversions). Events inferred as chromothripsis are shown in red and BFB in green).

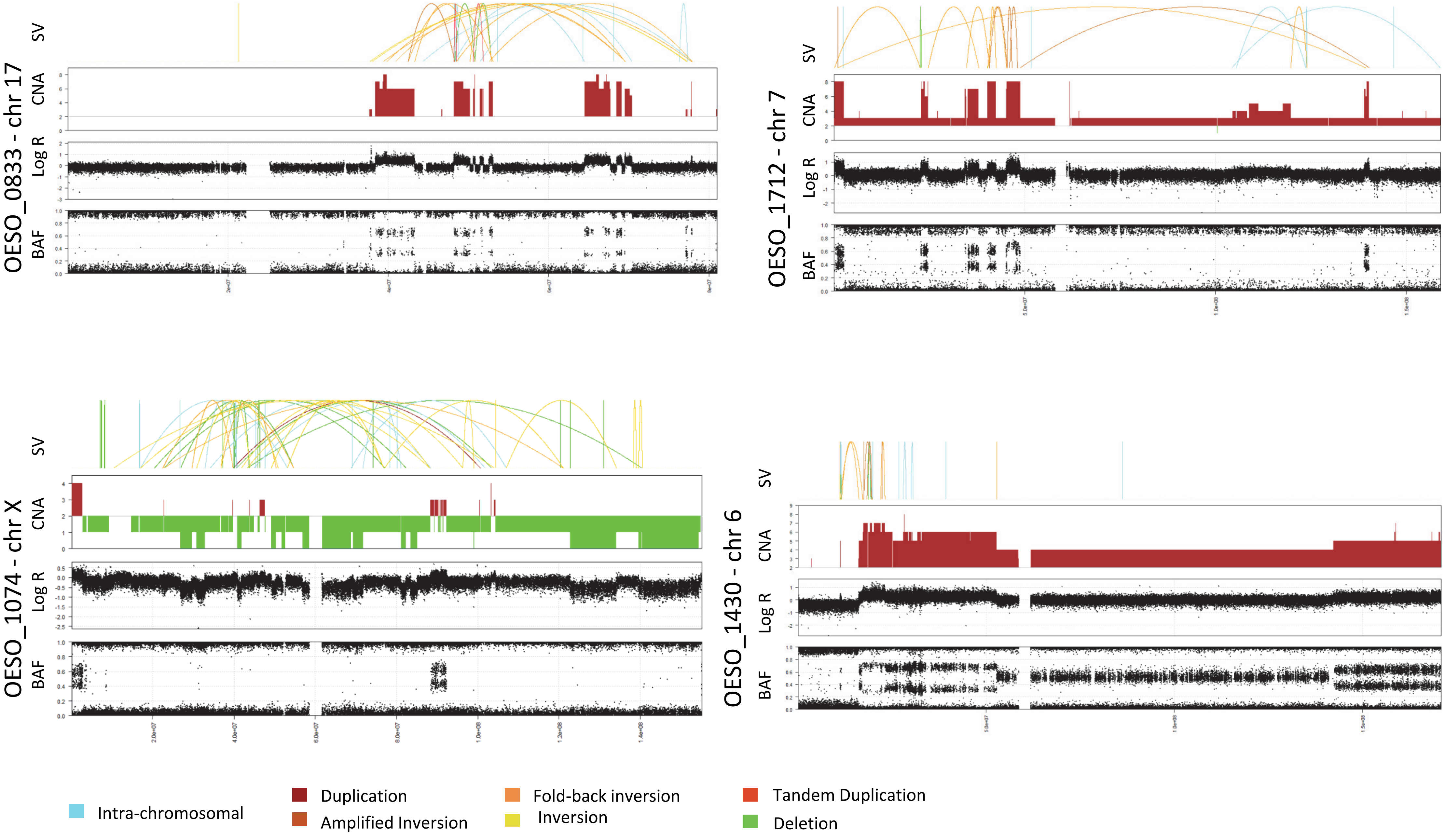

Supplementary Figure 6. Plots of chromosomes with inferred chromothripsis. Tumor id and chromosome is presented in each graph. Graphs show from the top SVs, copy number, logR ratio and B allele frequency.

OESO_0023 – chr10

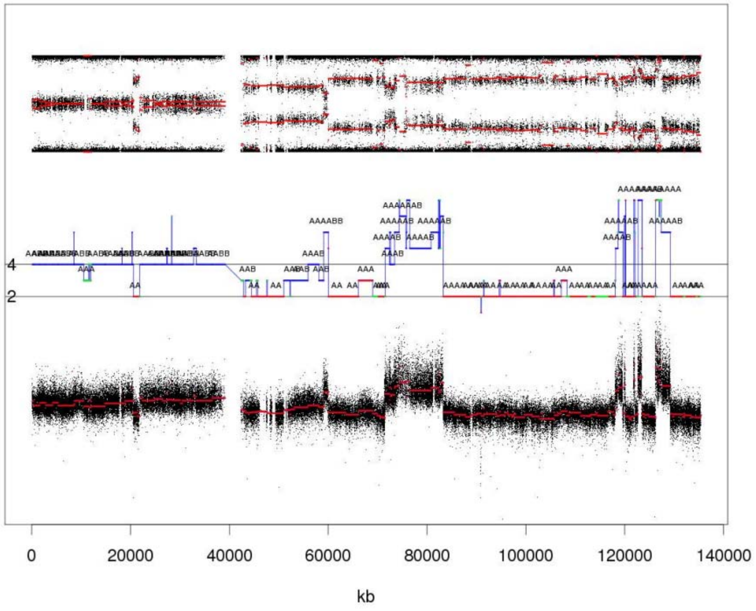

OESO_0031 – chr8

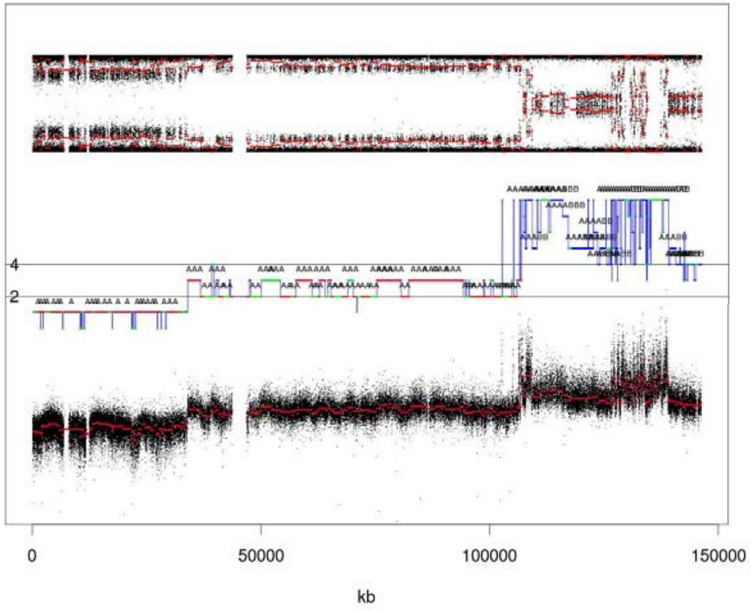

OESO_0052 – chr10

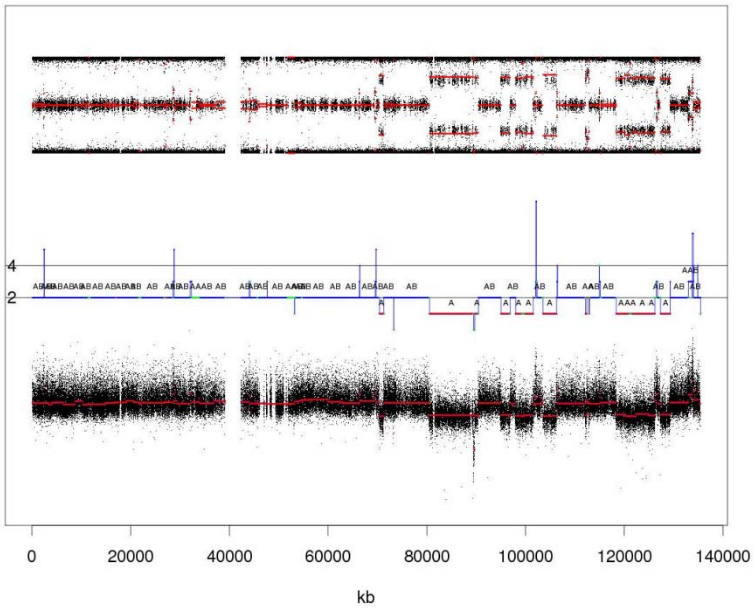

OESO_0053 – chr11

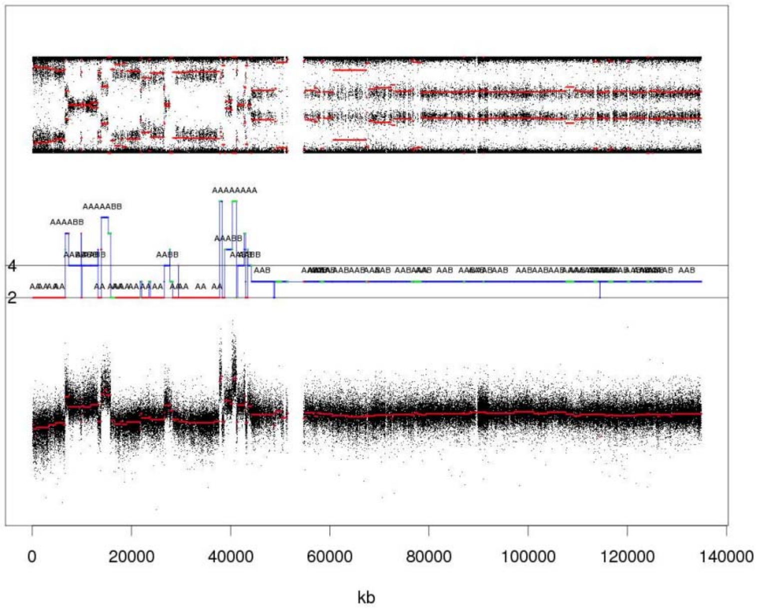

OESO_0008 – chr1

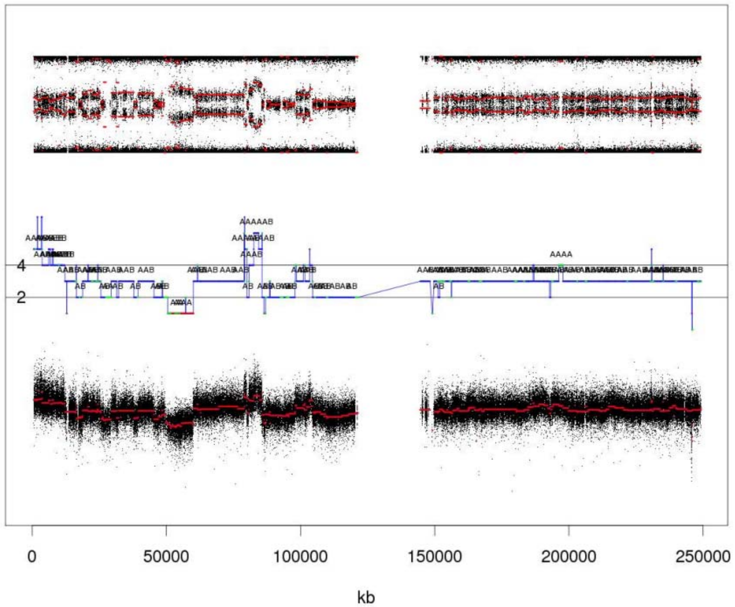

OESO_0010 – chr9

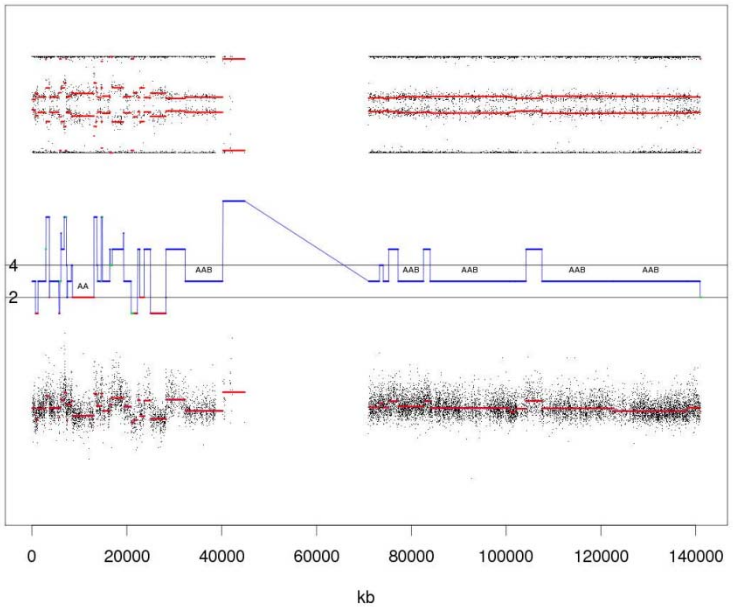

OESO_0015 – chr8

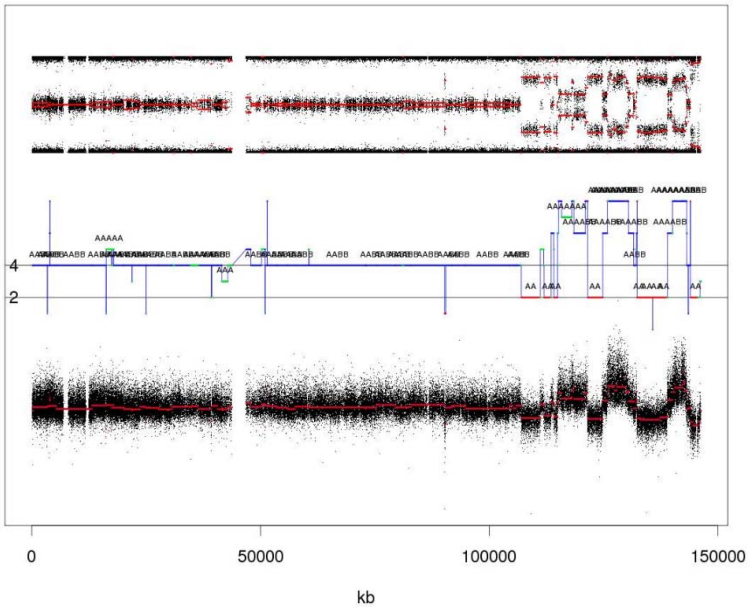

OESO_0021 – chr5

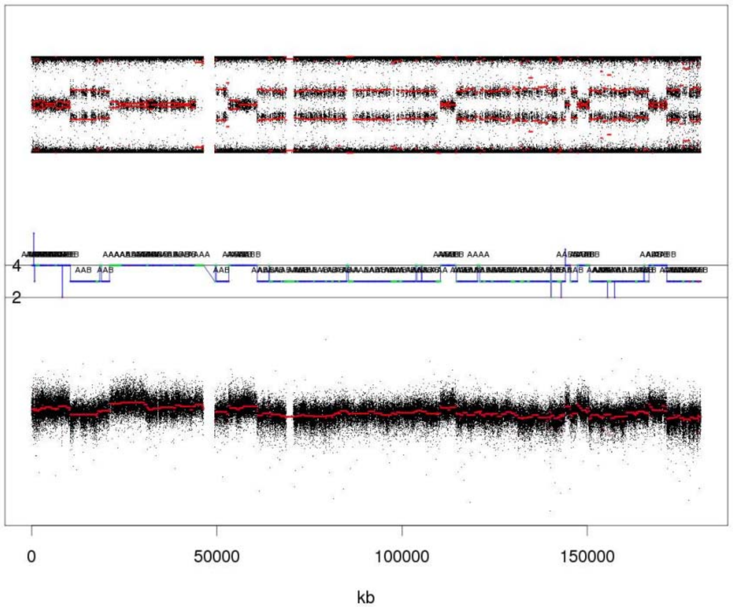

OESO_40325 – chr6

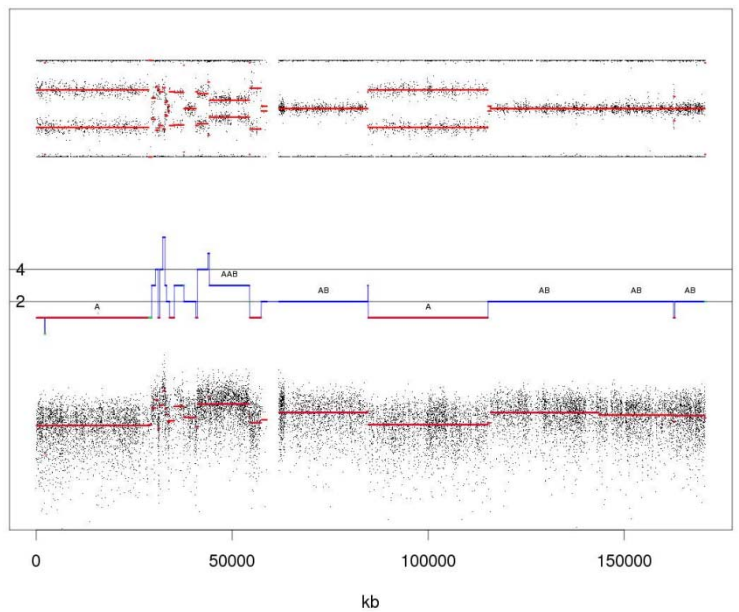

OESO_40328 – chr5

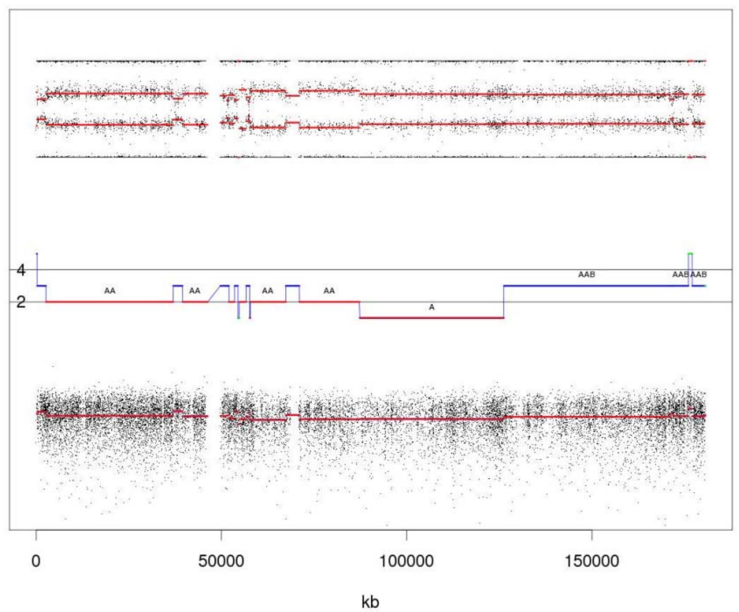

OESO_40357 – chr8

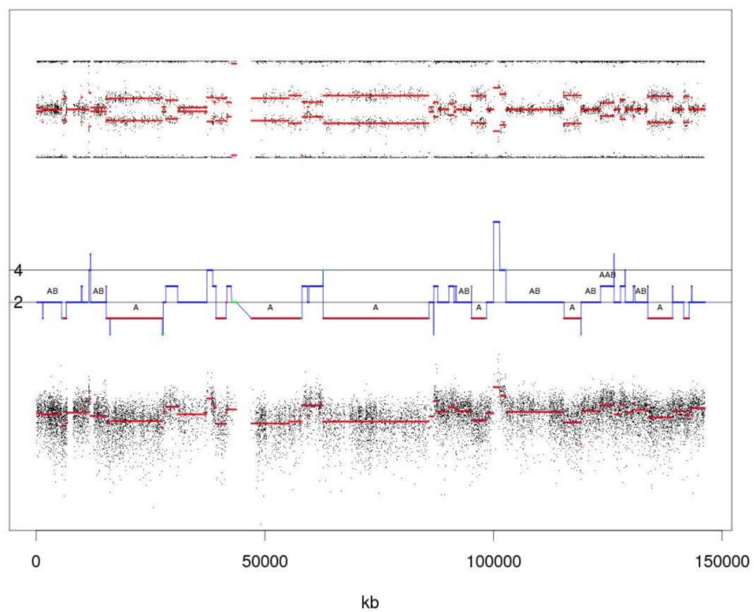

OESO_40336 – chr22

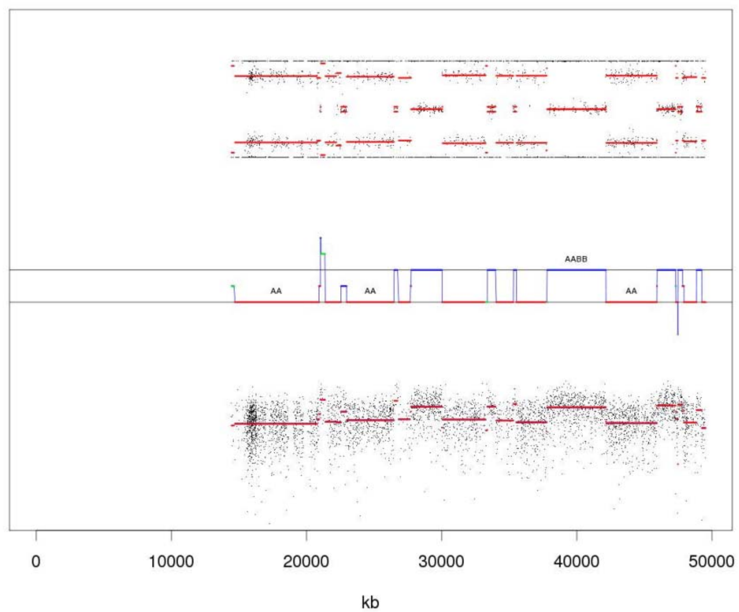

OESO_6003 – chr16

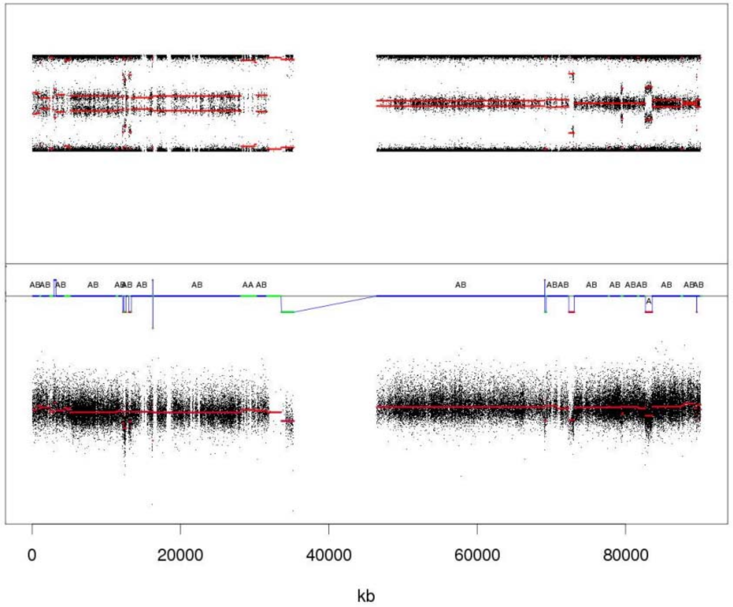

OESO_40364 – chr10

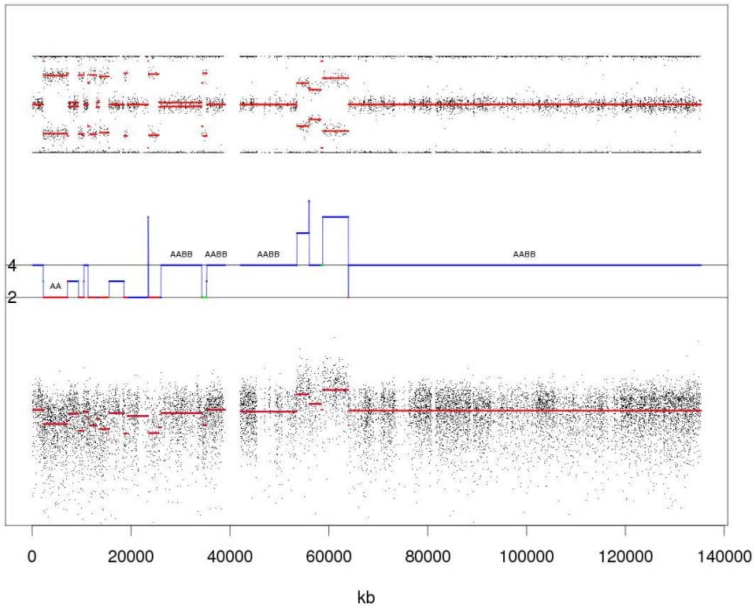

OESO_40365 – chr7

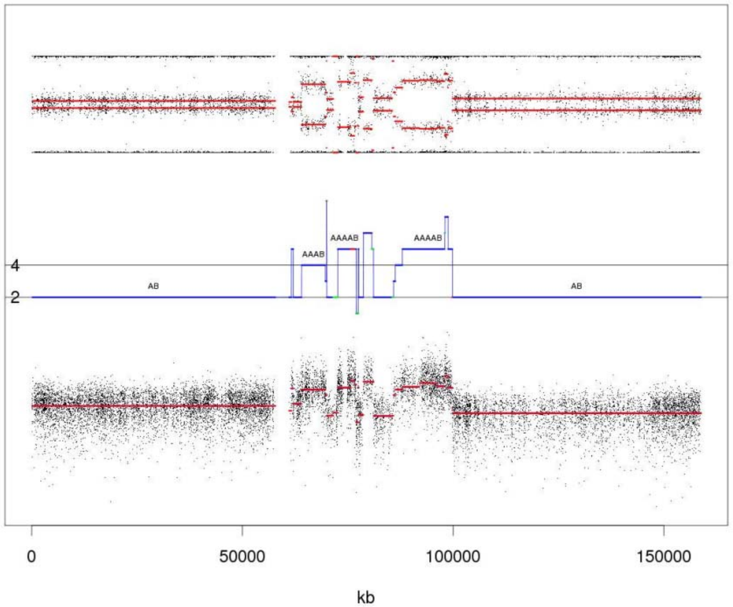

OESO_0001 – chr18

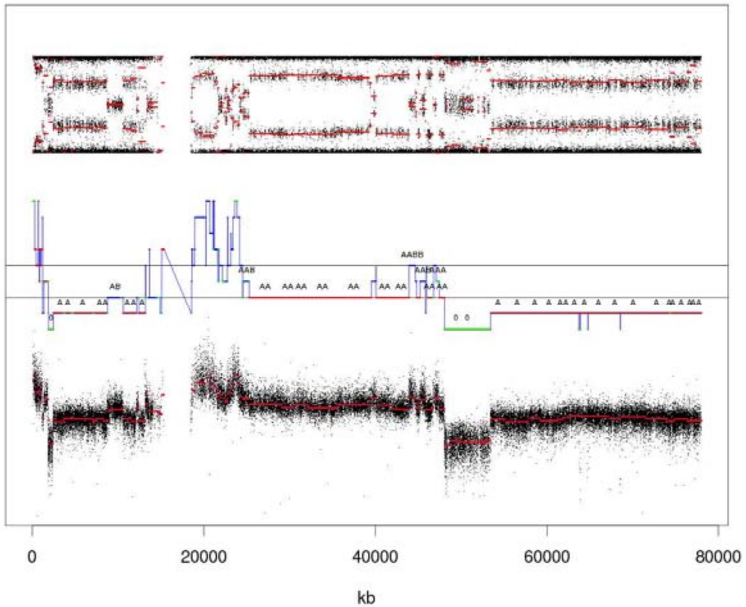

OESO_0056 – chr8

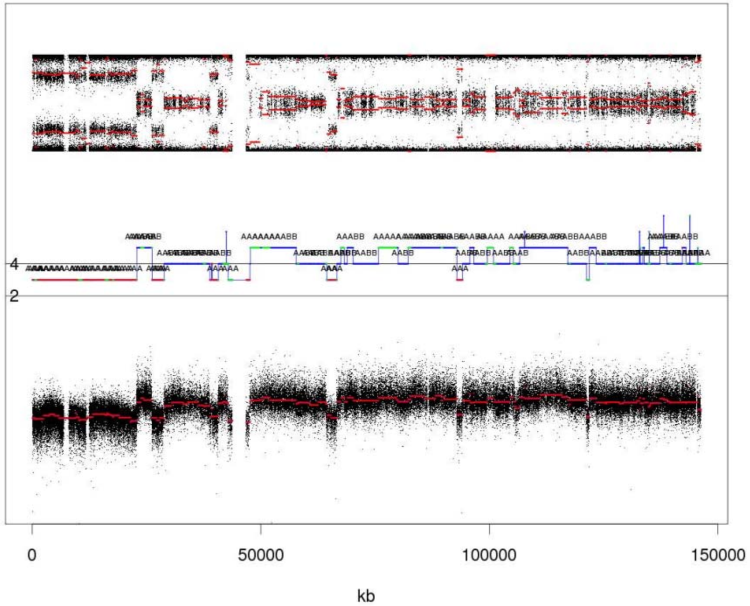

OESO_0061 – chr3

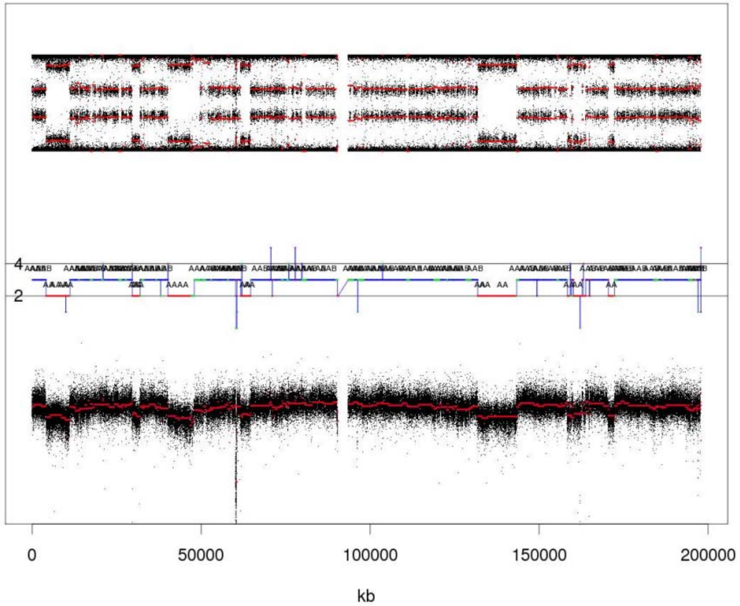

OESO_0063 – chr9

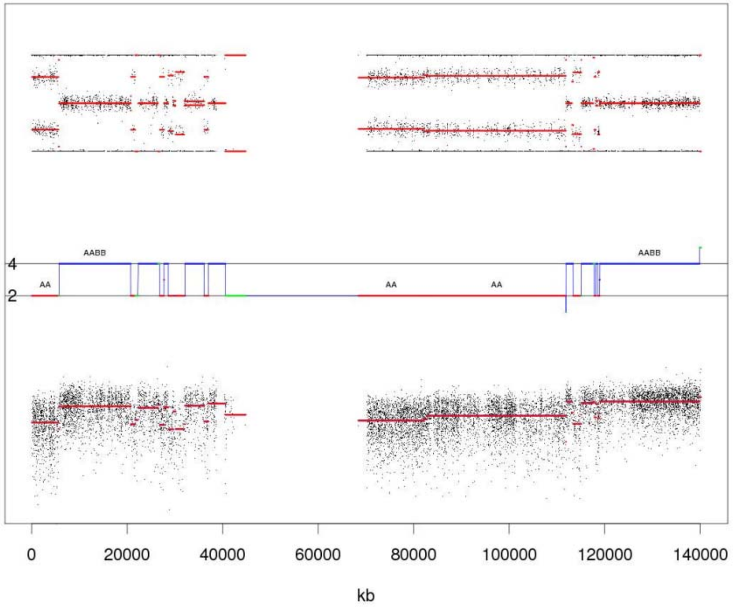

OESO_0572 – chr10

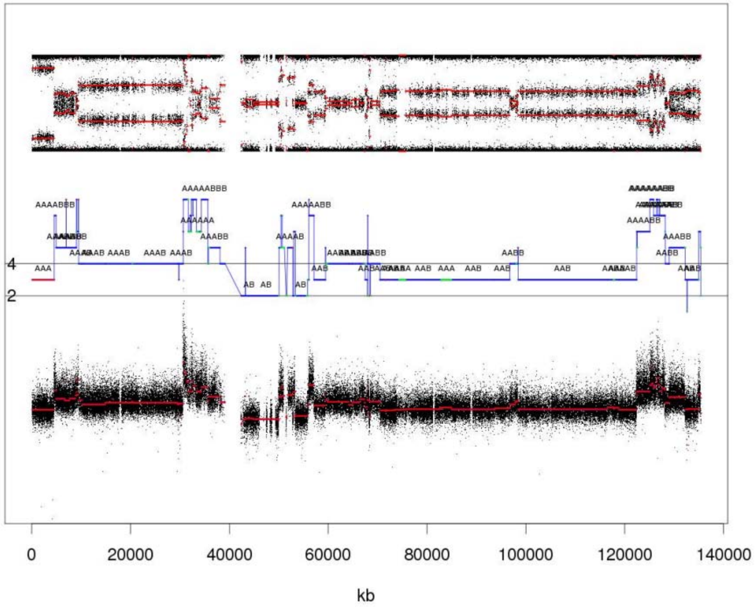

OESO_1642 – chr6

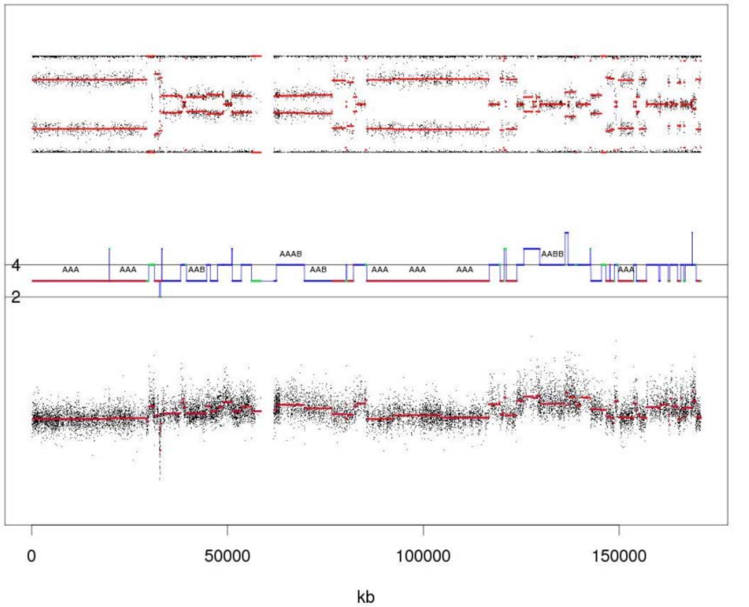

OESO_2442 – chr9

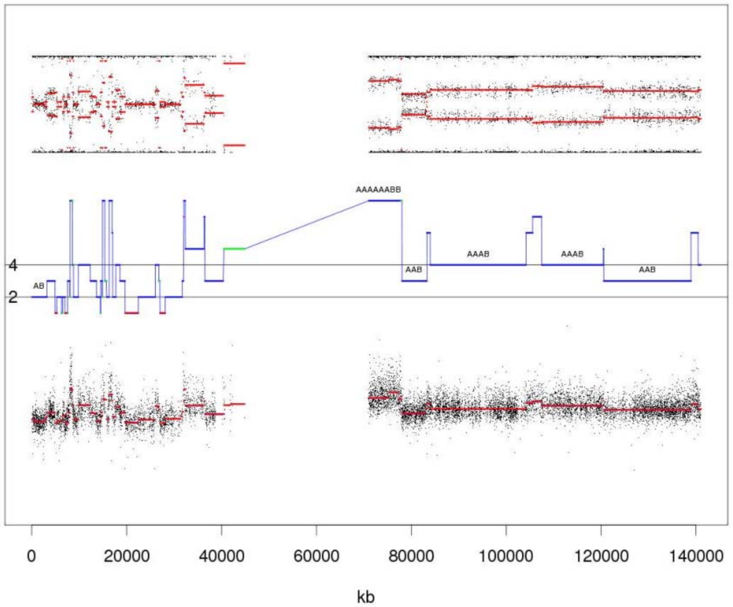

OESO_3519 – chr8

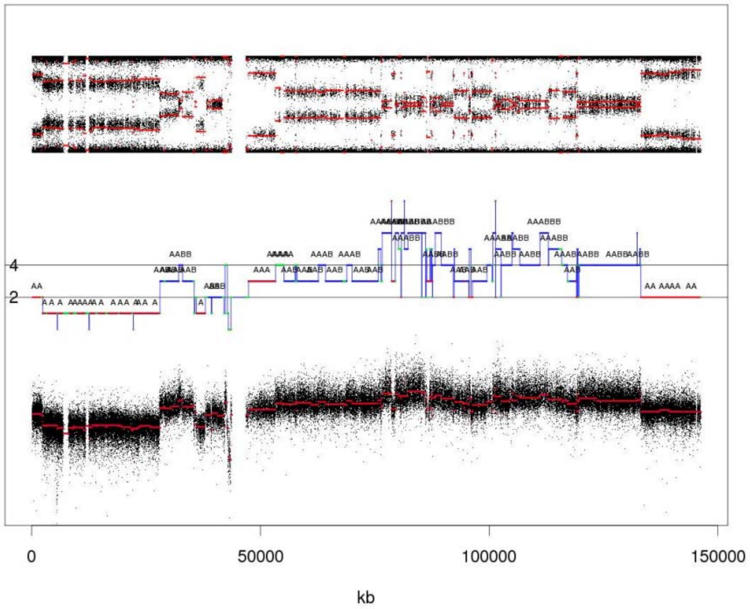

OESO_6005 – chr9

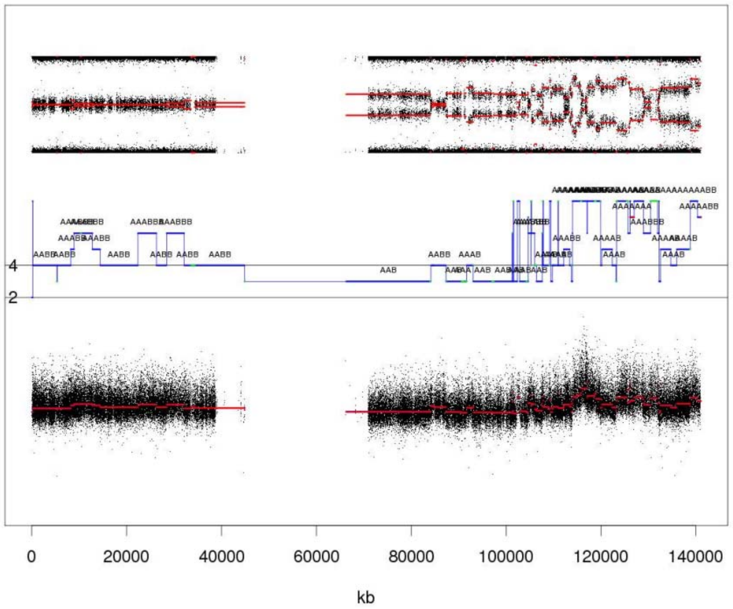

OESO_6036 – chr14

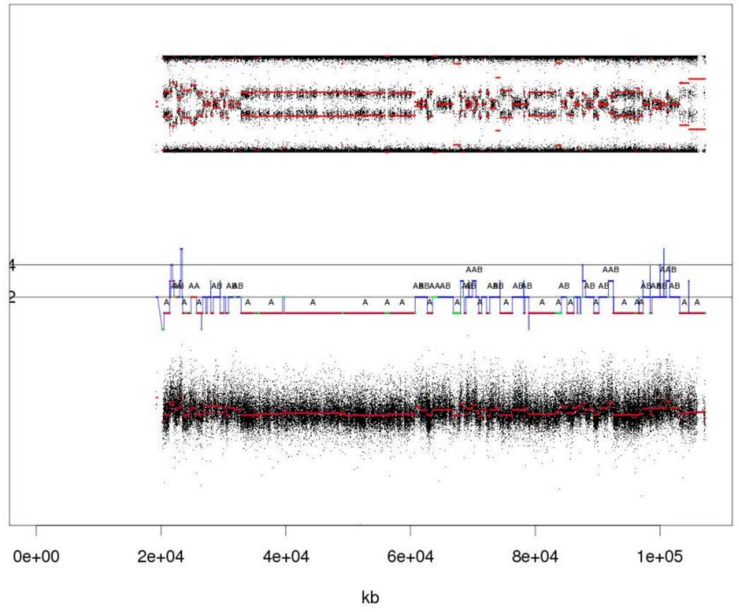

OESO_6039 – chr12

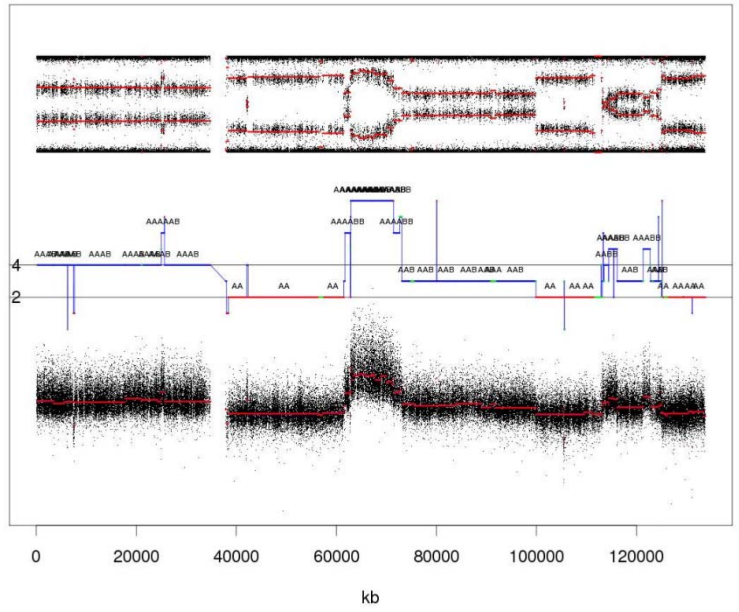

OESO_6040 – chr18

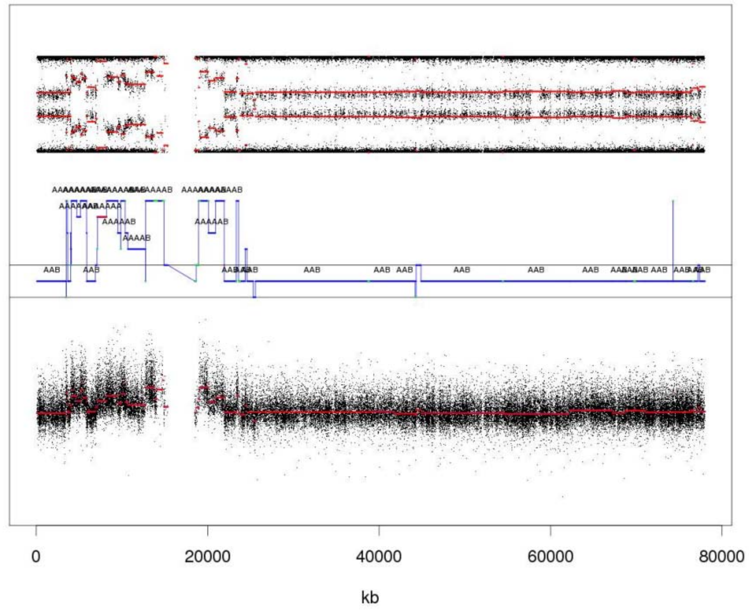

OESO_6052 – chr1

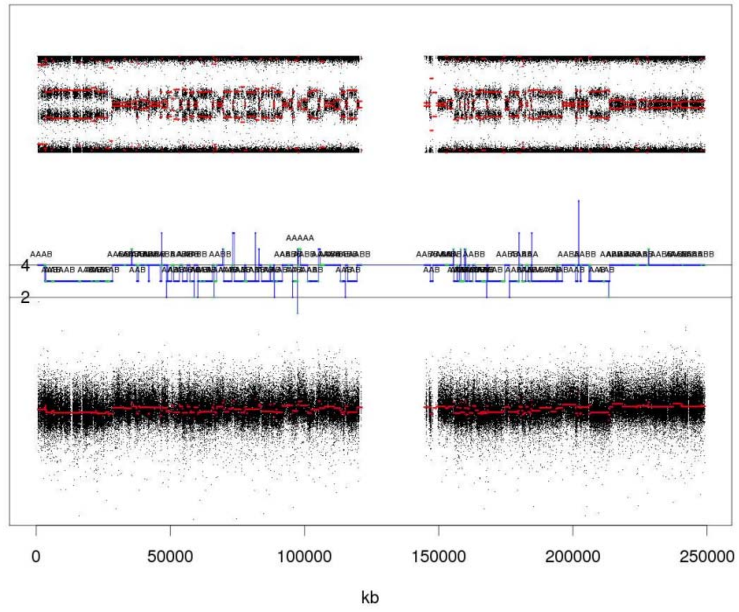

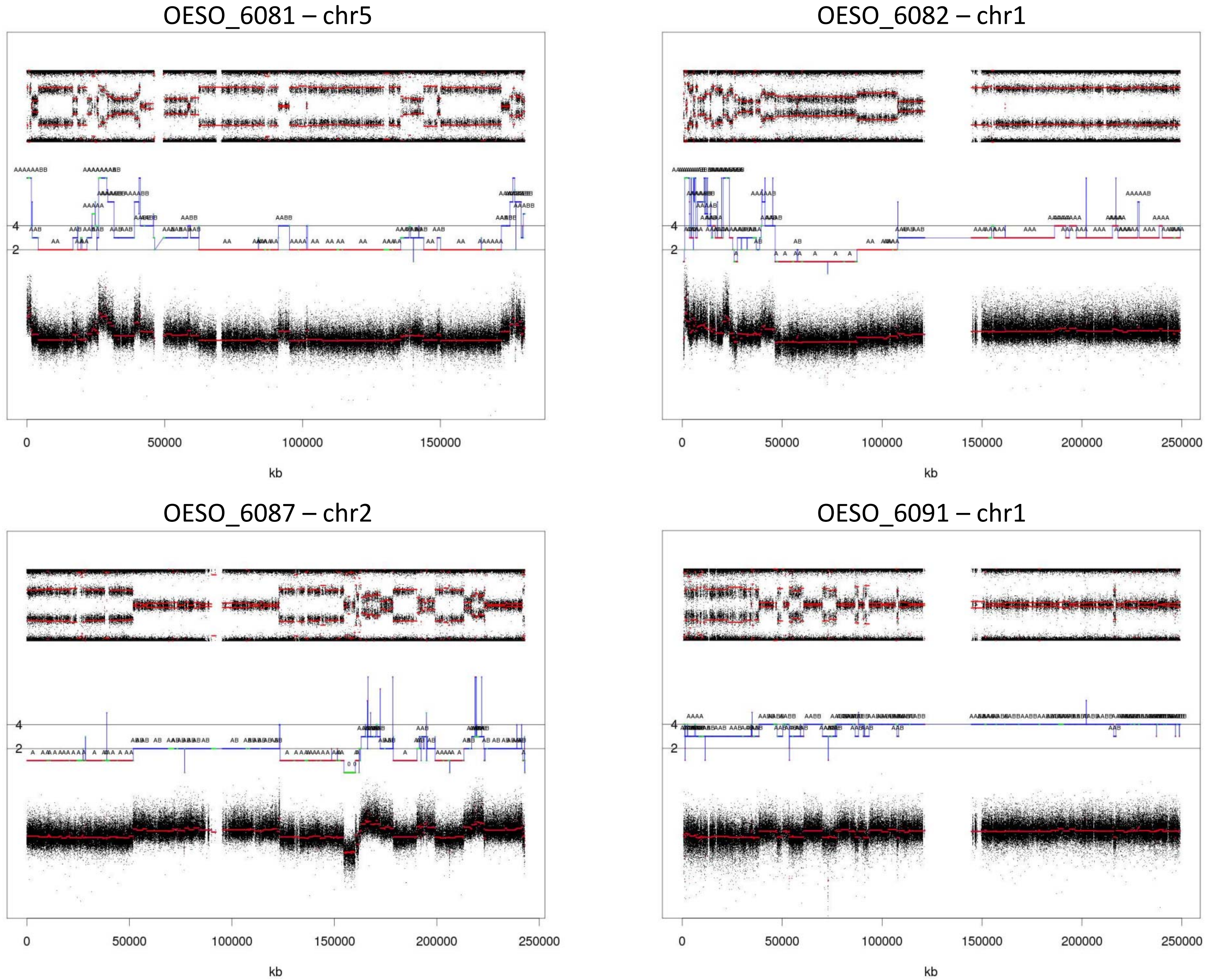

Supplementary Figure 7. Tumour with inferred chromothripsis showing ≥ 10 changes in segmental copy number in one or few chromosomes based on SNP array data. Plots show tumor id, chromosome BAF (top), copy number segments using GAP and Log R ratio (bottom).

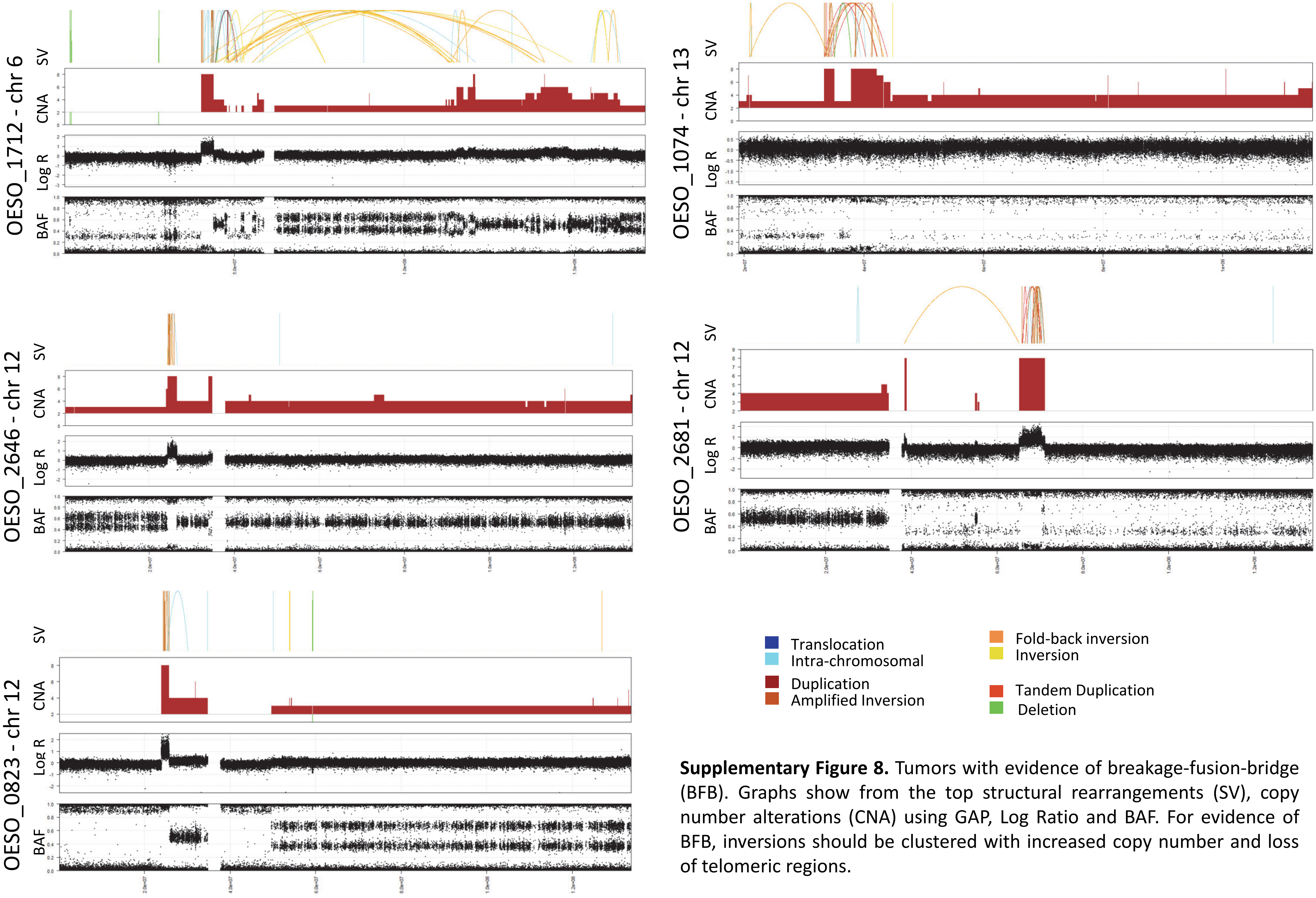

Supplementary Figure 8. Tumors with evidence of breakage-fusion-bridge (BFB). Graphs show from the top structural rearrangements (SV), copy number alterations (CNA) using GAP, Log Ratio and BAF. For evidence of BFB, inversions should be clustered with increased copy number and loss of telomeric regions.

a

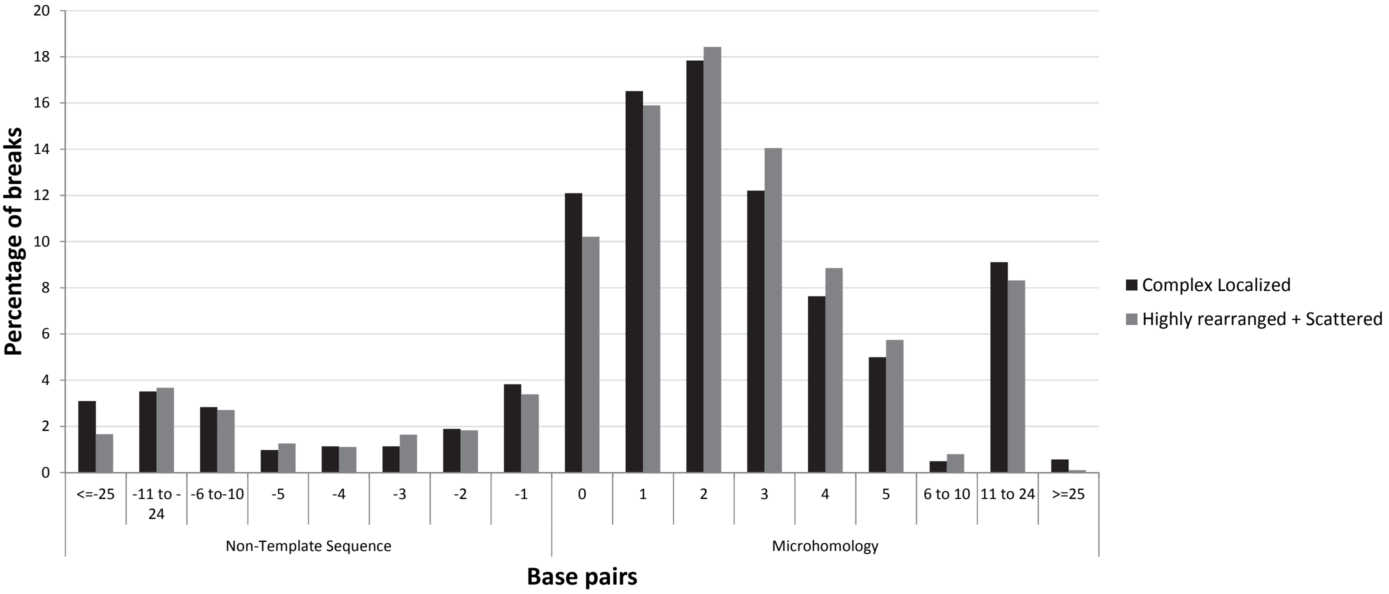

b

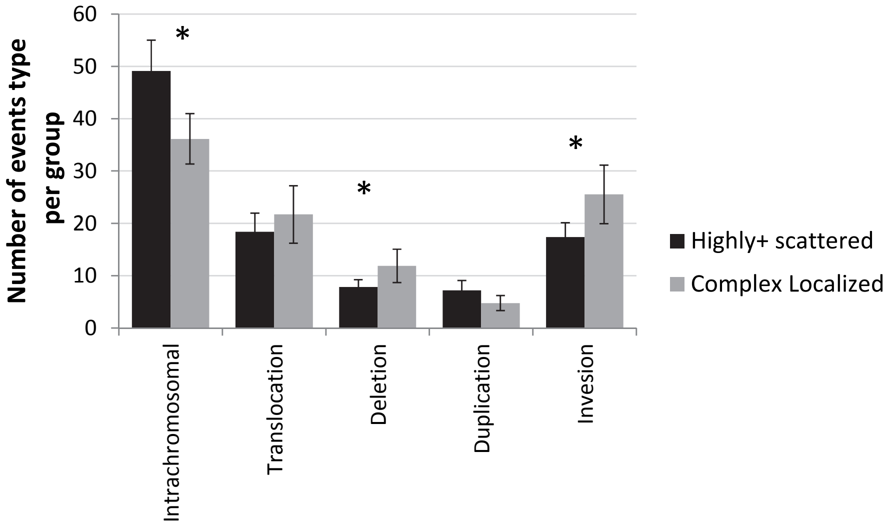

Supplementary Figure 9. Overview of SV types and breakpoint characteristics according with groups identified (based on SV numbers and distribution in the genome). a) Breakpoint characteristics in each group. Homologies in base pairs are shown as positive numbers. Blunt end has homology of 0 bp. Non-template sequences (small insertions) are shown as negative numbers. All tumors presented a higher percentage of breaks with 1 to 5bp microhomology. b) Show frequency of SV types in each group. Tumors with complex localized events harbor less intrachromosomal rearrangements and more deletions and inversions (*p-value≤0.05, t-test).

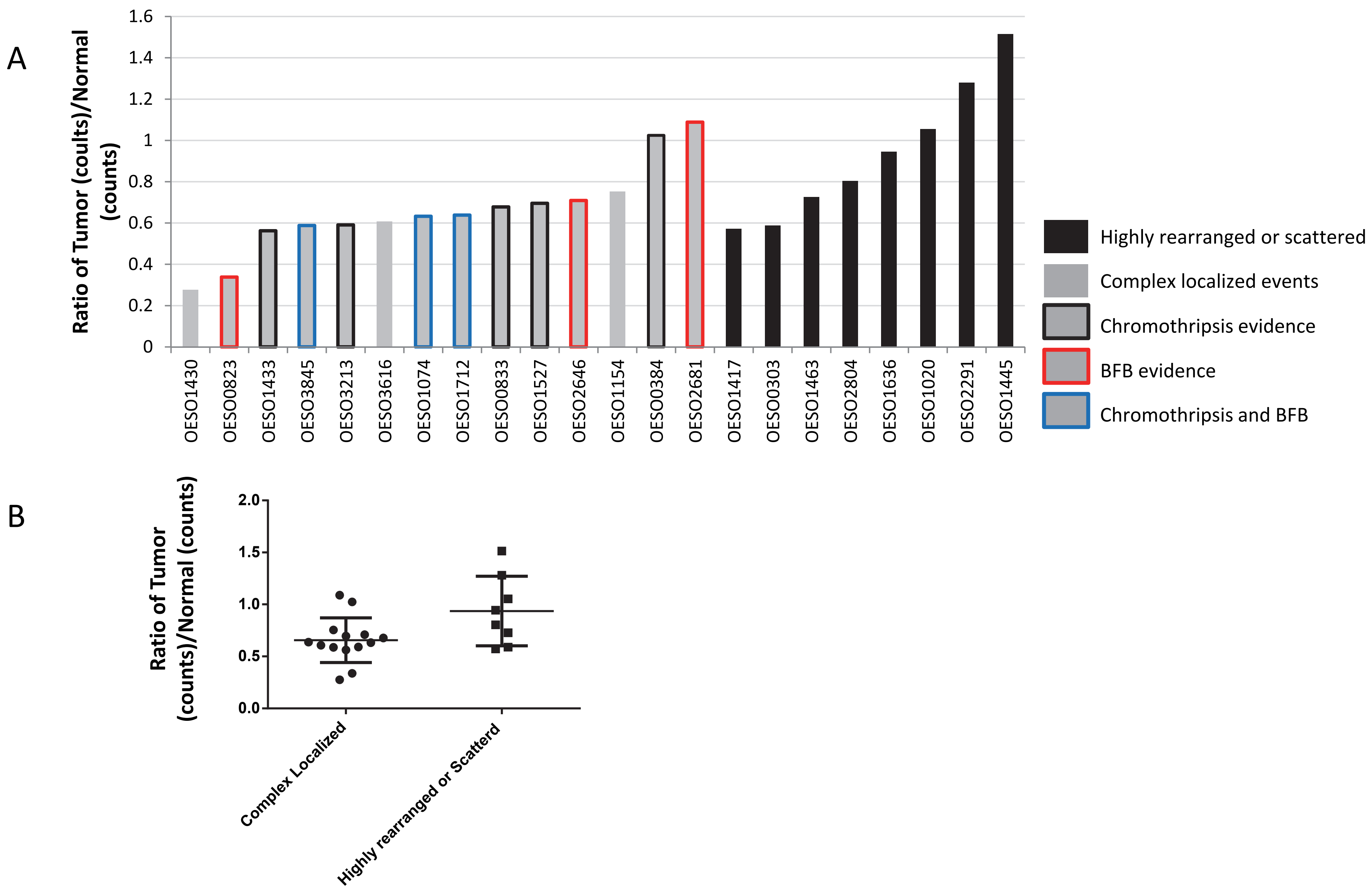

Supplementary Figure 10. Telomere analysis using whole genome sequencing data of 22 tumor and matched normal. Reads containing the telomeric repeat (TTAGGG)x3 or (CCCTAA)x3 were counted and normalized to the average genomic coverage. A) Ratio between tumor counts and normal counts. Ratio <1 suggest loss of telomere regions compared to the matched normal sample. B) Shortening of telomeres is most prominent in EACs bearing localized complex rearrangements (p=0.03, t-test).

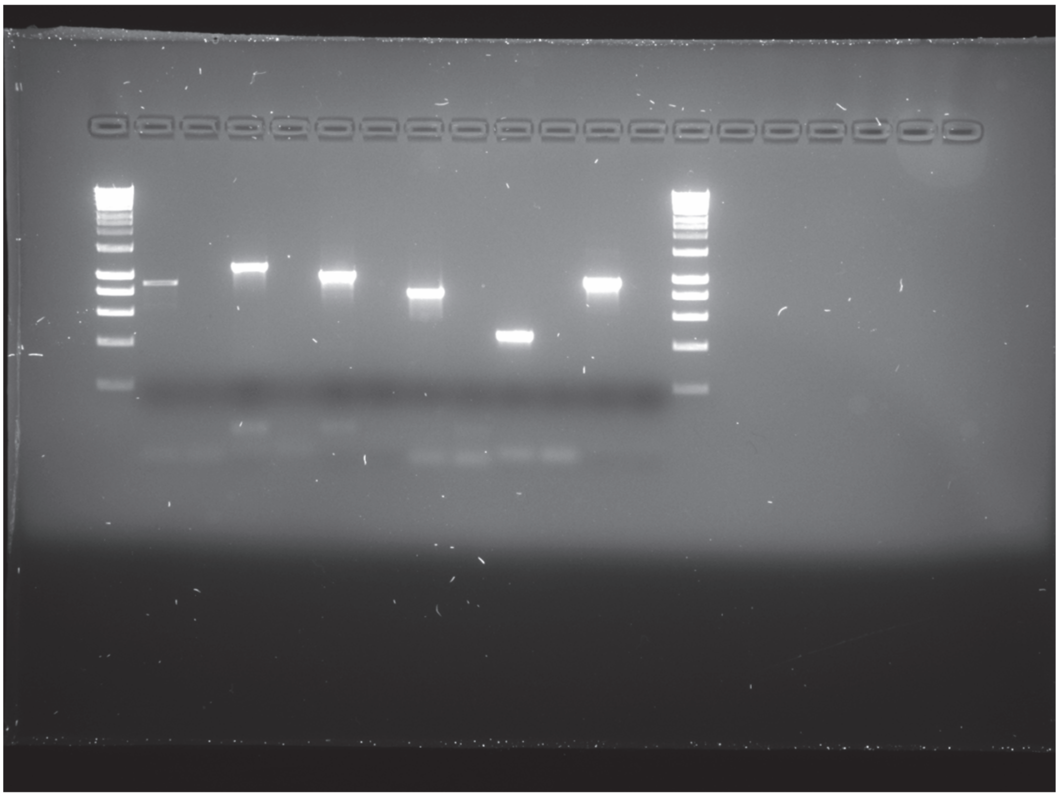

Supplementary Figure 11. Full Image of gel presented in the Figure 2f

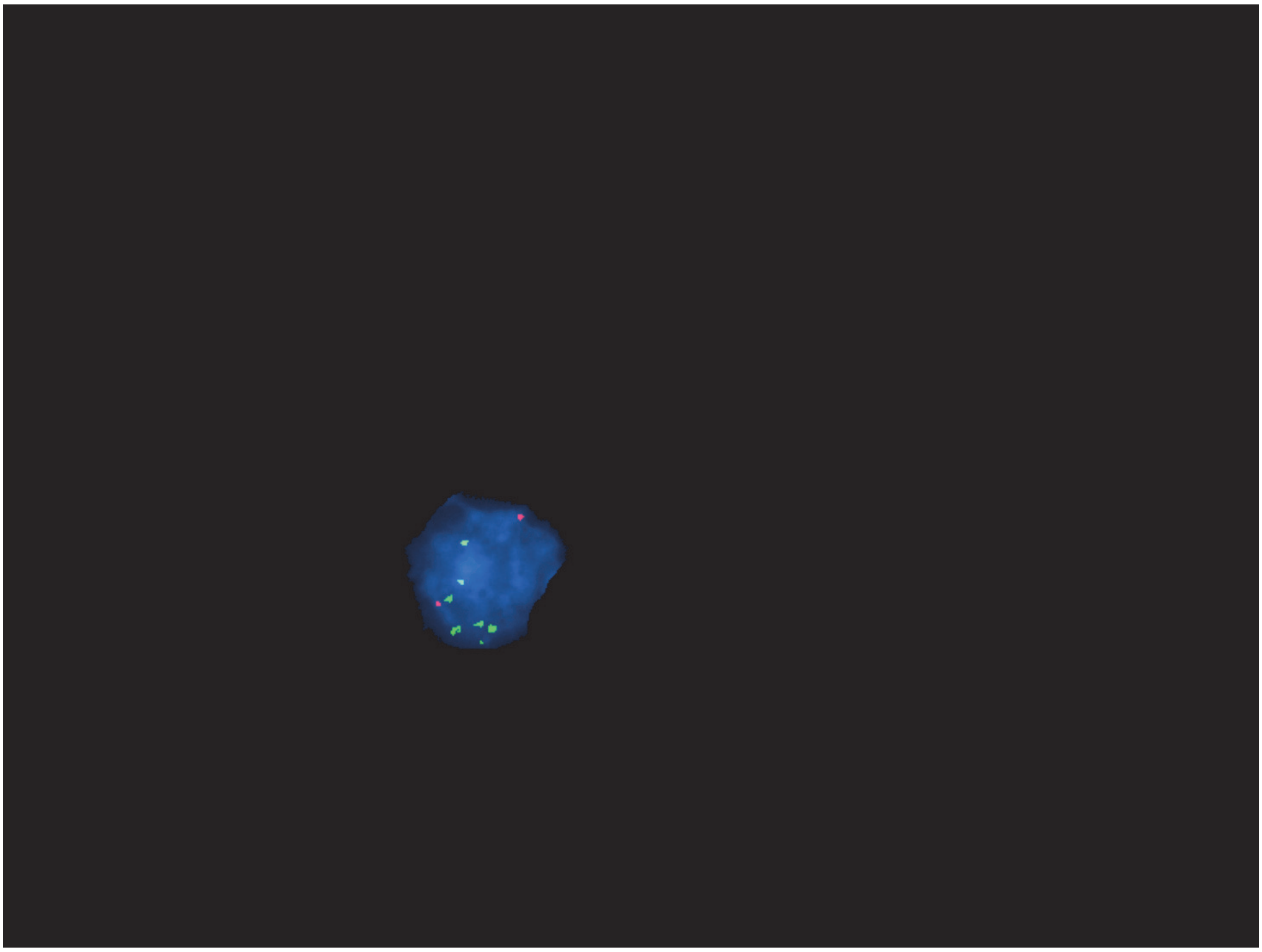

Supplementary Figure 12. Full Image of FISH presented in the Figure 2g

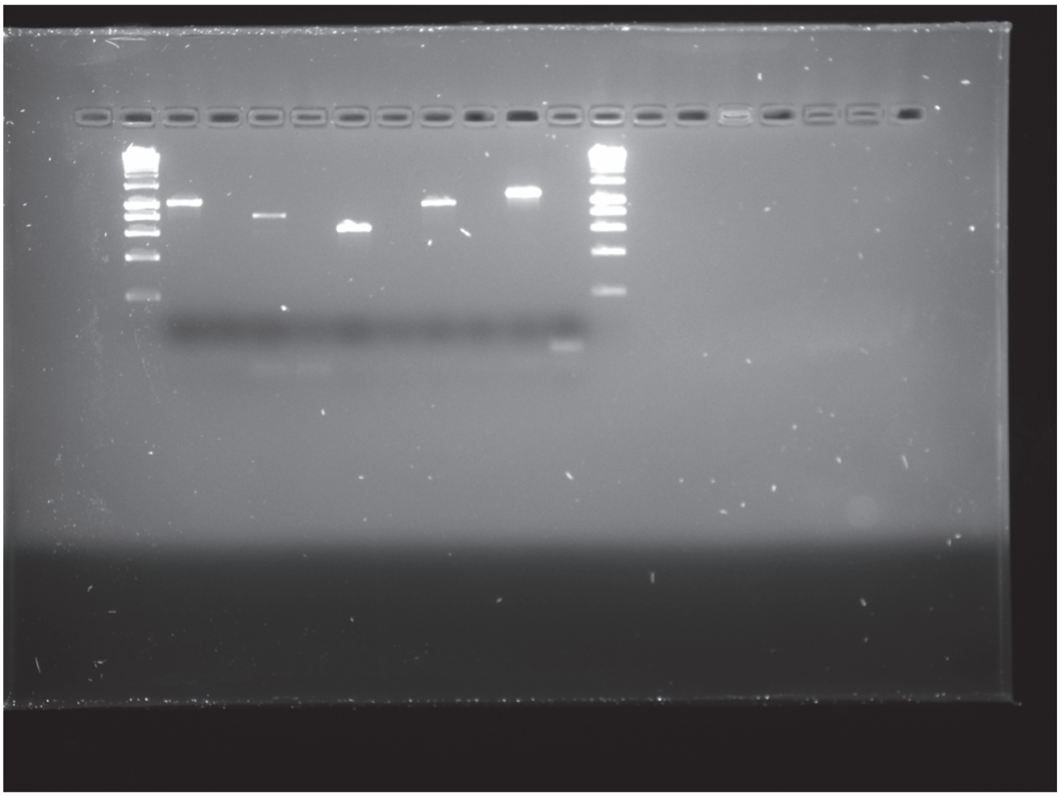

Supplementary Figure 13. Full Image of gel presented in the Figure 3d

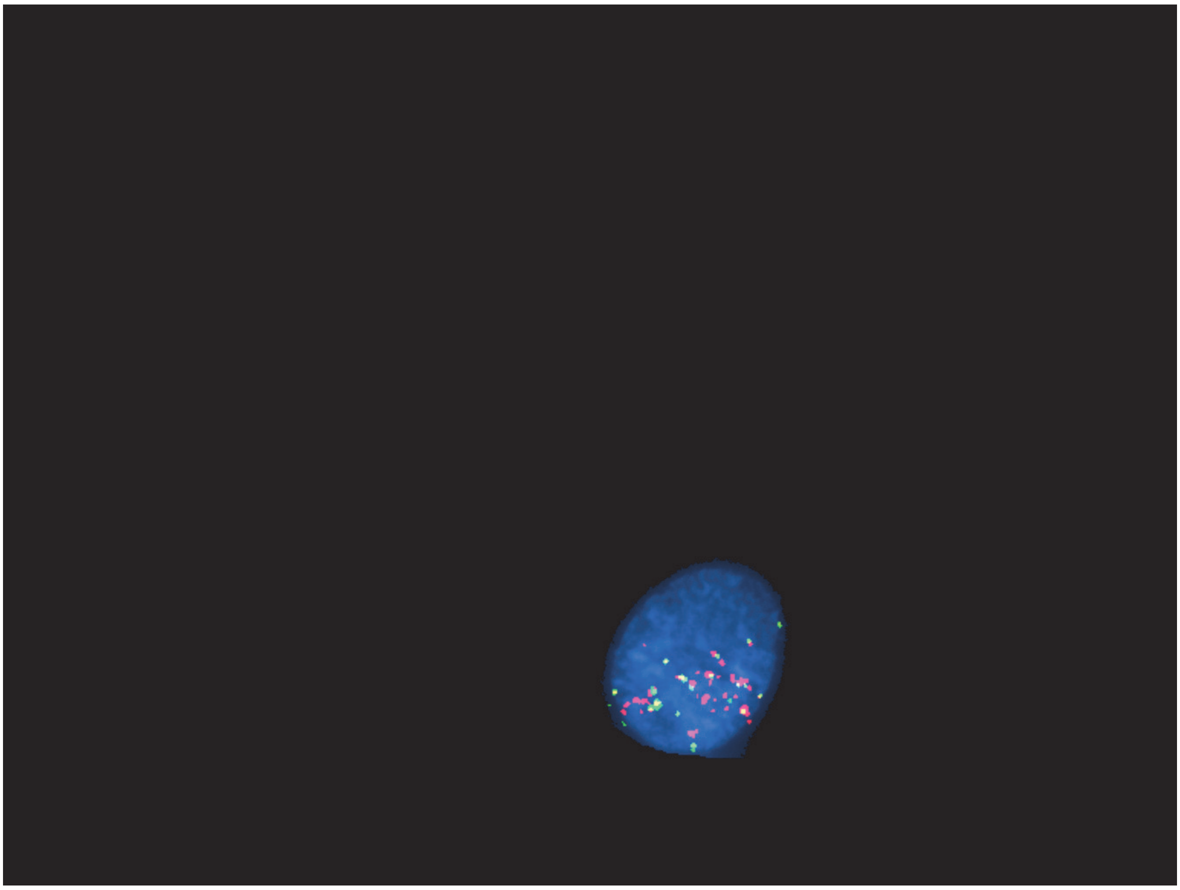

Supplementary Figure 14. Full Image of FISH presented in the Figure 3e

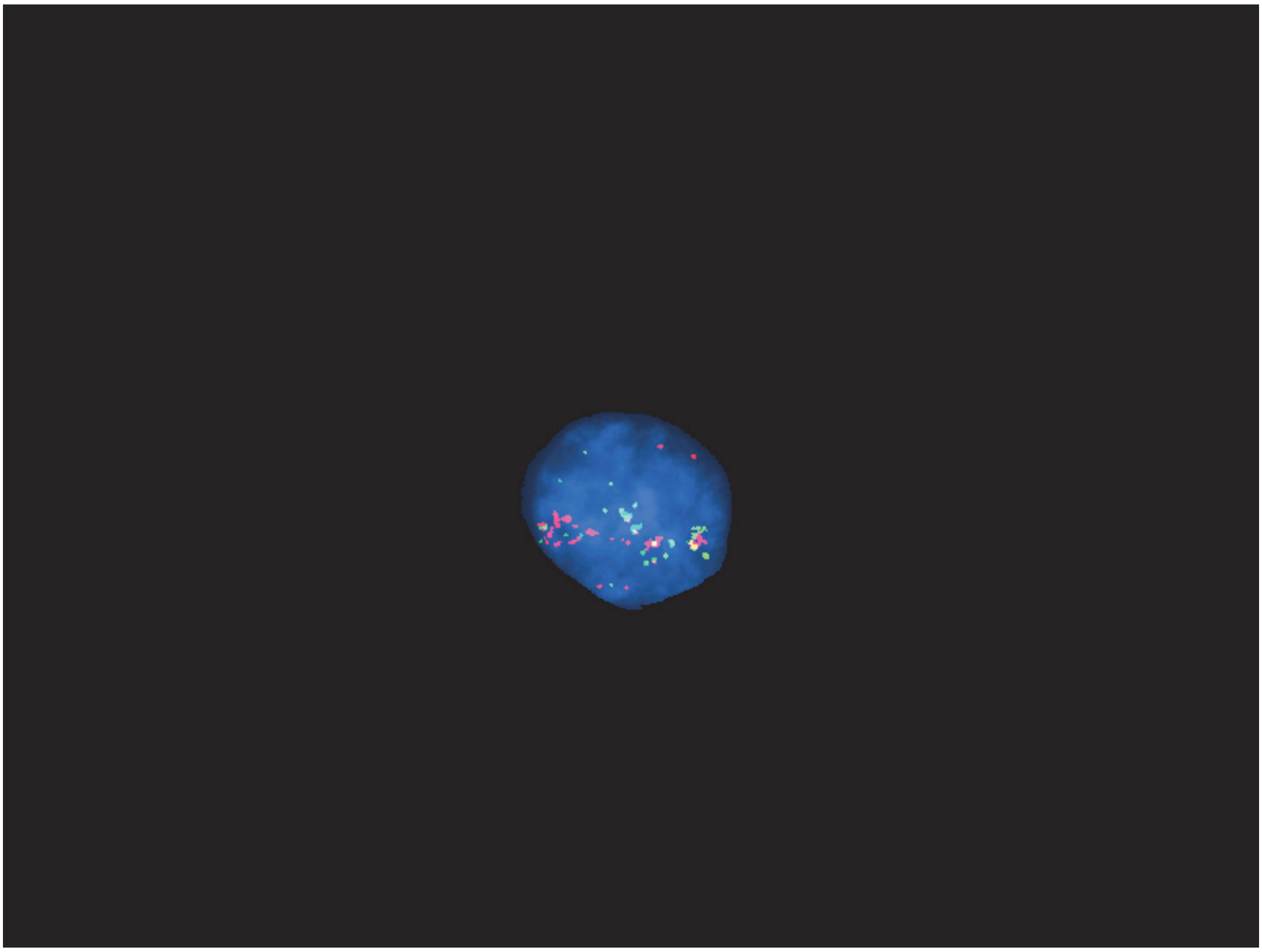

Supplementary Figure 15. Full Image of FISH presented in the Figure 3f

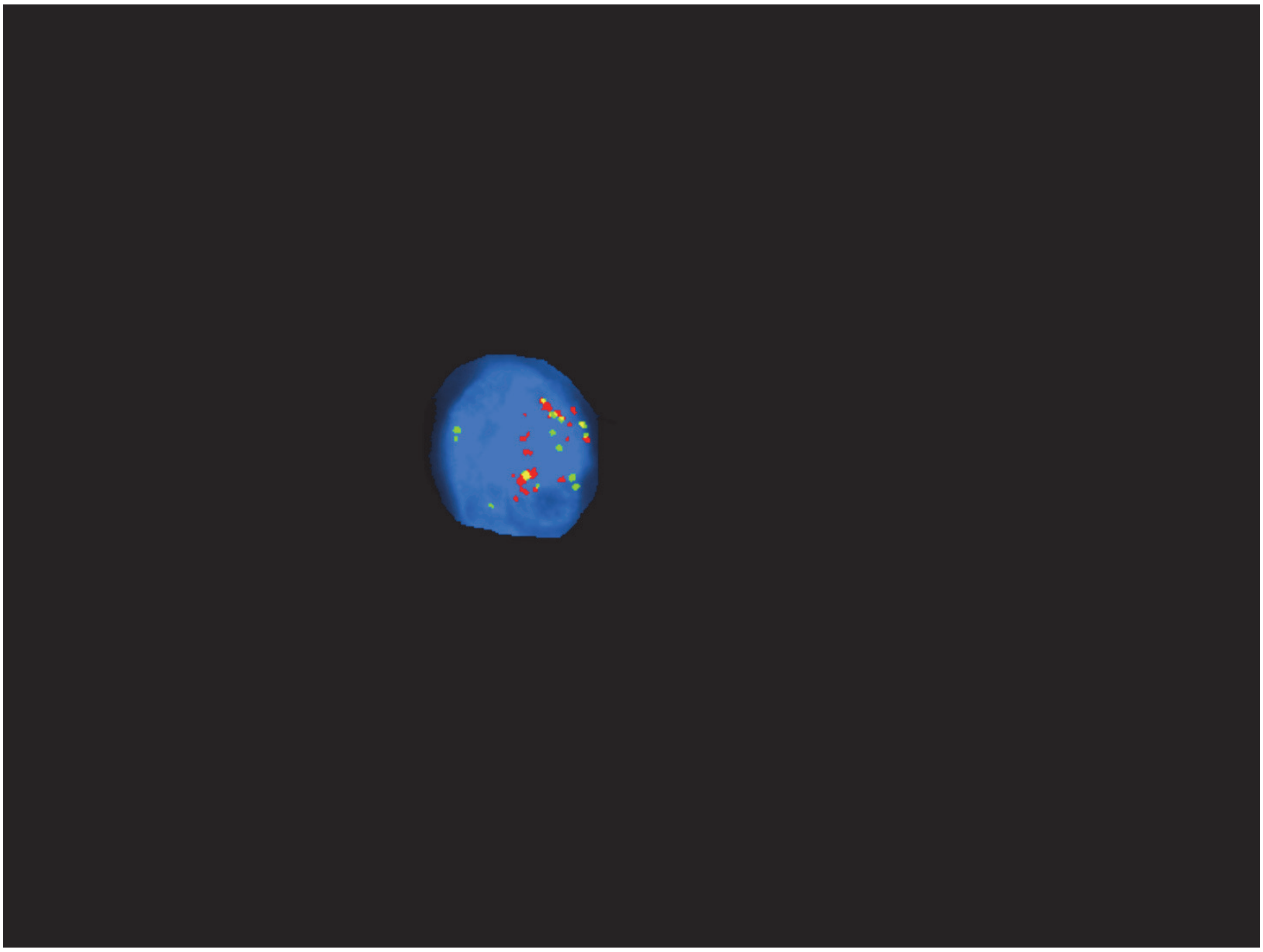

Supplementary Figure 16. Full Image of FISH presented in the Figure 3g
